# Back to the pre-industrial age? FAOSTAT statistics of food supply reveal radical dietary changes accompanied by declining body height, rising obesity rates, and declining phenotypic IQ in affluent Western countries

**DOI:** 10.1080/07853890.2025.2514073

**Published:** 2025-06-14

**Authors:** Pavel Grasgruber

**Affiliations:** Faculty of Sports Studies, Masaryk University, Brno, Czech Republic

**Keywords:** Child health, physical growth, nutrition, obesity, flynn effect

## Abstract

Meta-analyses of observational and clinical studies conducted in recent years have raised serious doubts about the validity of the low-fat dietary recommendations introduced in the late 1970s/early 1980s, due to the absence of any convincing link between saturated fat and the risk of cardiovascular diseases. At the same time, long-term food supply statistics from the FAOSTAT database show that these recommendations were at the root of fundamental dietary changes in Western countries, which resulted in a lower consumption of eggs and red meat, a higher consumption of cereals and poultry, a decline in average protein quality and, overall, in a higher glycemic load of the diet. Because current views on human nutrition are based primarily on highly unreliable questionnaire data from observational studies, the purpose of this commentary is to provide an alternative ecological (country-level) perspective and to trace the consequences of these nutritional changes using the FAOSTAT database in combination with available anthropological and health statistics. This comparison shows a close connection between the decline in protein quality and the sudden reversal of the positive height trend in some Western countries, after ∼150 years of continuous growth, which points to suboptimal levels of child nutrition. The sharp increase in the prevalence of obesity and type 2 diabetes is strongly correlated with the increasing consumption of high-glycemic carbohydrates and sweeteners, and is also interconnected with the decrease in body height, because a high-quality, growth-stimulating diet during adolescence is inversely related to obesity. Given the long-term association between height and phenotypic IQ, the lower quality of nutrients in children’s diet may also seriously affect intellectual potential and future civilizational development. In light of these findings, current nutritional strategies should be seriously reconsidered and recommended protein intakes for children must be urgently reevaluated.

## Introduction

**Figure 1.**
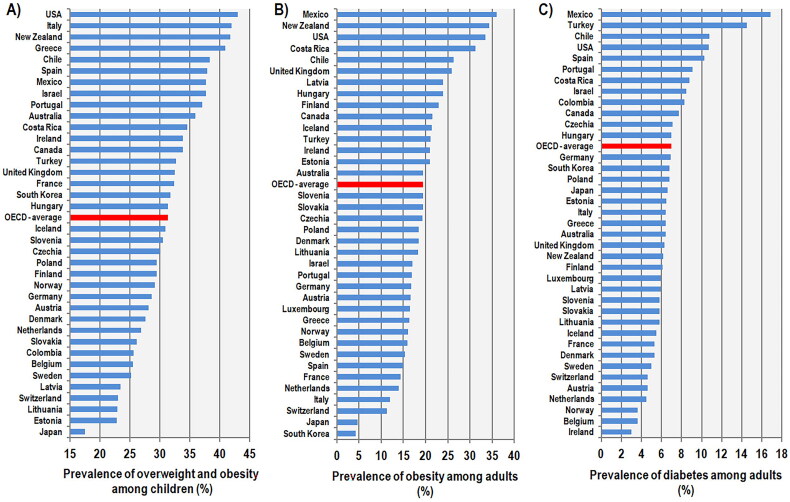
(A) Prevalence of overweight and obesity among children aged 5–9 years in 38 OECD countries (for 2016). Adapted from the OECD iLibrary [7], Health at a Glance 2019. (B) Prevalence of obesity (self-reported BMI > 30 kg/m^2^) among adults in 37 OECD countries (for 2021 or the nearest year). Adapted from the OECD iLibrary [7], Health at a Glance 2023. (C) Prevalence of type 1 and 2 diabetes among adults in 39 OECD countries (for 2021 or the nearest year). Adapted from the OECD iLibrary [7], Health at a Glance 2023.

**Figure 2.**
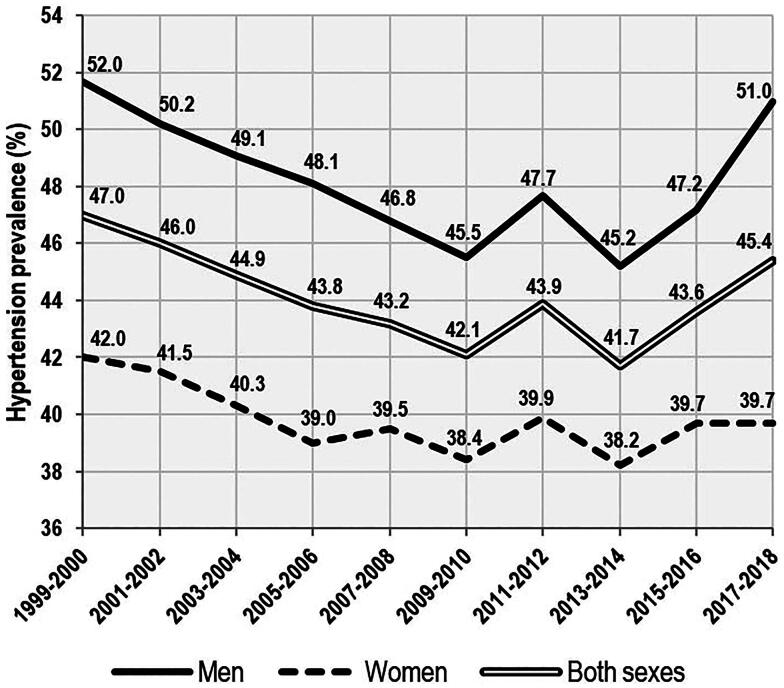
Age-adjusted trend in hypertension prevalence among adults aged 18 and over, by sex: United States 1999–2018. Hypertension is defined as systolic blood pressure greater than or equal to 130 mmHg, diastolic blood pressure greater than or equal to 80 mmHg, or current use of blood pressure-lowering medications. Adapted from the Centers for Disease Control and Prevention [8].

Over the last four decades, many Western countries have seen an alarming increase in obesity and type 2 diabetes, which has led to the intense enforcement of plant-based dietary recommendations based on a high proportion of whole grains, fruits, vegetables, nuts, and legumes. These dietary policies have further intensified in the context of the fight for ecological sustainability and against global warming [1,2], and have affected even children’s nutrition. For example, the British program Food for Life, which has been implemented in more than 7500 British schools [3], is based on a nutritional philosophy that calls for a reduction in meat consumption [4]. However, leaving aside the temporary impact of the COVID epidemic, the trend of increasing obesity in children aged 10-11 shows no signs of improvement [5]. In the USA, where the same view on this issue is shared, obesity rates vary by ethnicity, but the general situation is similarly dismal [6]. In fact, it is typically Anglo-Saxon and Mediterranean/Hispanic countries that dominate the OECD statistics of obesity and type 2 diabetes [7], whereas countries from Central and Northern Europe (particularly Belgium, the Netherlands, Sweden, and Switzerland) reach a relatively low prevalence (Figure 1A–C). In addition, the most recent data from the National Health and Nutrition Examination Survey (NHANES) [8] show that the US population is experiencing a reversal of a long-term downward trend in the prevalence of hypertension, indicating an increasing risk of cardiovascular diseases (Figure 2). This finding confirms the existence of a chronic health crisis that is unlikely to improve in the near future.

The main thesis of this commentary is that this situation is not only a consequence of civilizational bad habits in the form of reduced physical activity but also of radical nutritional changes. These stem from nutritional recommendations issued in the late 1970s and have been further exacerbated by the increasing consumption of ultra-processed, nutritionally poor foods. Long-term trends in food consumption, which can be derived from statistics in the FAOSTAT database, reveal that these dietary changes are manifested in the form of declining protein quality, increasing carbohydrate consumption, and increasing glycemic load of the diet. By comparing these data with available anthropological and health statistics, it is possible to reach a justified conclusion that these shifts in eating habits not only have negative effects on the overall health of the adult population but also negatively affect the physical and mental development of children, which can potentially have far-reaching consequences for the entire society.

## The use of the FAOSTAT database in nutrition research

Relationships between health and human nutrition are traditionally investigated through observational (cohort) studies in which the participants fill in self-reported information about food consumption in questionnaires. However, it is understandable that such self-reported data, collected once every few years, suffer from many inaccuracies and biases, and are increasingly perceived as pseudoscientific [9,10]. For change, controlled clinical trials – which are the only studies capable of reliably tracing a causative effect – are of very short duration. As a result, the lack of accurate, long-term information on food consumption is responsible for all the confusion in nutrition science. A typical example is the different relationship between animal foods and health across different continents – clearly detrimental in the United States, less evident in Europe, and inconsistent, often beneficial in East Asia [11–13]. These discrepancies indicate that the results of nutritional studies can be influenced by geographically specific confounders or official state policies.

Due to these limitations, our research group tested the practical usability of the food balance statistics collected by the FAOSTAT database [14], which contains data on the per capita supply (availability) of >100 food items or food groups since 1961. These statistics are computed by combining the total quantity of foodstuffs produced in each country with the total quantity imported and are further adjusted to the quantities exported, fed to livestock, used for seed, put into manufacturing for food use and non-food uses, and losses during storage and transportation. The obtained amount of each food item that is actually available for human consumption is then divided by the country’s population. The calculated per capita averages include four major ‘elements’: food supply quantity (kg/per capita/year), food supply (kcal/per capita/year), protein supply quantity (g/per capita/year), and fat supply quantity (g/per capita/year). Carbohydrate supply is not available but can be estimated by subtracting energy from proteins (4.1 kcal/g) and fats (∼9 kcal/g). The methodology of this database somewhat changed in 2014 [15] and two versions (1961–2013 and 2010–) are currently available. Originally, the differences between these two versions were rather negligible, but the new version has been further modified after the addition of the year 2021. Although the shape of trend lines remained practically identical, absolute values in the period 2010–2013 are sometimes markedly different, and the two versions should only be combined with caution [16].

It is reasonable to assume that even after all sophisticated corrections, the FAOSTAT data may not reflect well the average consumption at the individual level because a significant part of the available food is wasted, consumed by domestic animals or, for example, by tourists visiting the countries in question. However, the practical experience of our research team is different and we have documented many impressive relationships between the per capita supply of certain foods and the prevalence of selected disease indicators. The strongest of these was the correlation between the prevalence of raised cholesterol (for 2008) and the average supply of animal fat & animal protein (1993–2008) in 42 European countries (*r* = 0.92 in men, *r* = 0.88 in women; *p* < 0.001) [17], which is an expected finding because the most potent dietary trigger of raised cholesterol is saturated fat [18], whose primary source in the human diet is animal food. A subsequent study examining the environmental causes of cancer [19] likewise demonstrated many strong correlations as high as *r* = 0.80, supporting present-day views on causal factors, some of which (such as the link between tea drinking and esophageal cancer) have been put forward only very recently. Very convincing results can also be obtained from a comparison of the average supply of high-quality proteins and population averages of body height (see below).

Based on this experience, it can be concluded that the FAOSTAT statistics produce valid findings and reflect relative inter-country differences in food consumption with a high degree of accuracy. This can be explained by the fact that the possible confounding factors operate more or less equally in all countries. The usefulness of the FAOSTAT database has been verified in practice by many other researchers and it is generally recommended that the most accurate results are achieved by averaging long-term data or by expressing them relatively, as trends or ratios [20]. Naturally, it can be expected that the accuracy of these statistics is lower in less developed countries, but this is not relevant for the purposes of this text.

In a nutshell, the practical results of ecological studies contradict the categorical opinion of Walrabenstein et al. [21], who argued that ‘aggregated [ecological food supply] data cannot be attributed to the individual level’. Despite the fact that these statistics only represent population averages and cannot account for inter-individual differences in dietary patterns, it is evident that in the examples mentioned above, the average dietary pattern corresponds to the average biological effect. In fact, meaningful relationships can be found even in cases where large sex-related differences in food consumption can be expected (e.g. alcohol drinking). Thus, despite their country-level design, ecological comparisons still provide very useful clues for examining health-related phenomena at the level of individuals.

Although it is understandable that individually collected data from observational studies are considered more meaningful and in the past, ecological results were often downplayed because of the risk of ‘ecological fallacies’ (basically, the lack of information on hidden causal variables), it is important to realize that self-reported food consumption in observational studies often bears little relation to reality and is strongly confounded by healthy user bias. In addition, observational studies examine intra-population differences in food consumption, which are routinely too small to demonstrate a significant effect indicating true causality [22]. In contrast, modern ecological studies can utilize a wide range of reliable statistics from online databases, which can already cover almost the complete spectrum of possible causal factors and show impressively linear relationships across world populations with fundamentally different dietary habits. Also noteworthy is the legitimate assumption that inter-country differences in some hard-to-quantify lifestyle factors (e.g. physical activity, stress) are likely to be smaller than at the level of individuals. It is a curious paradox of contemporary nutritional science that top scientific journals reject ecological studies based on long-term, high-quality statistics but routinely publish observational studies with biologically impossible input data and highly confounded results that lack any reasonable explanation. This situation creates a vicious circle of ‘observational fallacies’ that further support flawed dietary strategies [23]. From this point of view, ecological studies represent a very valuable methodological alternative. Although they have similar limitations to observational studies (i.e. uncontrolled interactions among variables that require careful interpretation), comparing their results with observational studies can identify potential discrepancies that would merit special attention in nutrition science.

In this context, it is noteworthy that ecological findings based on the FAOSTAT database are in complete contradiction to the assumption that cardiovascular diseases (CVDs) are caused by high saturated fat intake – an enduring element of current nutritional recommendations. Factor analyses performed with the above-mentioned sample of 42 European countries [17] identified two major dietary styles typical of Mediterranean and Germanic-speaking countries, respectively, which showed a strongly negative relationship with CVD mortality and CVD prevalence (raised blood pressure, raised blood glucose), and whose common denominator was the high consumption of total fat and (animal) protein. These two dietary patterns also shared a high intake of high-fat dairy and fruits (especially citrus fruits), which had the most antagonistic relationship with CVD risk. Other important food items with a potentially positive impact include sources of plant fat (olives, tree nuts) and fish & seafood. On the other hand, indicators of cardiometabolic diseases were most strongly associated with dietary carbohydrates (mainly in the form of high-glycemic cereals and potatoes). The role of meat in these comparisons is essentially neutral or passively beneficial (as the source of fat and protein), and something similar can be said about low-glycemic plant foods (legumes, vegetables).

## The changing paradigm in human nutrition

This seemingly contradictory finding is not surprising when viewed from the perspective of current meta-analyses of observational studies and clinical trials, which have failed to find a convincing link between saturated fat and CVDs [24–26]. On the other hand, an association between CVDs and high carbohydrate intake/high dietary glycemic load is one of the most consistent findings found in observational studies [11,27–31], although it manifests persuasively only in women. Remarkably, this sex-specific discrepancy occurs even in the ecological analyses performed by our research group and is not difficult to explain because CVD risk in men is influenced by other negative lifestyle factors (especially higher smoking rates and alcohol drinking).

The very origin of the ‘saturated fat hypothesis’ in nutrition science can be traced to the ecological ‘Seven Countries Study’ which analyzed the statistics of coronary heart disease (CHD) in seven selected countries and whose interim results were used as an argument for the introduction of the low-fat recommendations in the United States (1977) [10,32,33]. However, these measures were not convincingly supported by contemporary clinical trials [34] and were rather the result of a hasty political decision. Although the fundamental flaw of the Seven Countries Study is attributed to the biased selection of the countries examined [10] or to its ecological design [33], it is obvious that the period CVD statistics suffered from many contradictions resulting from poor data collection, misdiagnosis, and inflated CVD death rates in affluent countries (due to the eradication of other causes of death and the sudden increase in life expectancy). These issues can be demonstrated by the fact that stroke mortality in the Seven Countries Study showed completely opposite correlations than CHD mortality [35]. Contradictions of this kind would be a clear signal that the entire research is fundamentally flawed, but they were discovered too late.

All of these issues were not resolved until the 1990s and 2000s, as evidenced by the increasing positive correlation between CHD and stroke mortality statistics, and the greatest irony is that current ecological data in Europe point very strongly to high carbohydrate intake as the key trigger of CVD [17]. Therefore, it is very likely that the effort to find a causal link between saturated fat and CVDs will be futile. In fact, current dietary guidelines targeting saturated fat are based mainly on four methodologically problematic clinical trials from the 1960s [10] or on observational studies performed in the United States, where the risk of CVDs is usually related to ‘red meat’ – a heavily confounded proxy for the unhealthy fast-food lifestyle of lower social classes [23] and peculiarities of the American diet [36]. At this point, it should be noted that controlled clinical trials have shown a mostly neutral or beneficial effect of red meat on key CVD risk indicators (HDL cholesterol, blood triacylglycerols, blood pressure). Red meat performs noticeably worse only when compared to diets containing fish [37,38]. Similarly, the growing body of evidence pointing to the beneficial effects of dairy products [39], which is independently supported by ecological findings, presents a fundamental conflict with the saturated fat hypothesis because milk fat contains twice as much saturated fat (∼60%) than meat and eggs (∼30%).

Recently, a new call for urgent changes in nutritional guidelines has come from an international team conducting the PURE study, which examines the relationship between disease incidence and diet in 21 countries around the world [40]. Although food consumption data in this project are also collected using questionnaires, the broad spectrum of countries included enables to trace different eating habits in a global context and it is also largely free of potential biases resulting from the fact that health-conscious people in Western countries follow official nutritional recommendations. Based on extended data from 80 countries, the authors identified high carbohydrate consumption as the central problem of human nutrition and proposed a ‘healthy PURE diet’ consisting of whole-fat dairy, fish, fruits, nuts, raw vegetables, and legumes, which has the most consistent protective effect against CVD on all continents [41]. Unprocessed red meat (and unprocessed meat in general) showed a negative association with CVD risk and all-cause mortality, which became neutral after multiple adjustments. In contrast, a negative health impact was observed for processed meat, which is certainly no surprise.

The results of this study have been criticized for potential shortcomings common to all observational studies, with poverty and malnutrition cited as the most likely confounding factors [42], but apparently, they are not difficult to reconcile with the ecological results mentioned above. At the very least, they show how dramatically the conclusions of observational studies can differ when conducted outside the developed Western world, where public opinions on ‘healthy’ and ‘unhealthy’ foods are influenced by state health policy.

Interestingly, from the view of the PURE study, the composition of the widely promoted ‘Lancet Planetary diet’ (which contains a relatively high proportion of carbohydrates and restricts animal products) [2] appears to be the least beneficial out of all other ‘healthy diets’ that are currently promoted worldwide, with no effect on the improvement of global health. In addition, legitimate objections have been raised that strict implementation of this diet would put large populations at risk of nutritional deficiencies [43]. The practical possibility of implementing such nutrition plans also has its limitations [44].

Nevertheless, it is important to emphasize that the recommendations of the PURE study would primarily benefit adults with low or moderate physical activity, and there is no ‘ideal diet’ for all population groups. Individuals with higher protein requirements (children, athletes, hard physically working people, pregnant women, physically fragile seniors) need to increase the proportion of food with high protein quality (cf. [45]). This crucial factor is often overlooked, leading to the uncritical promotion of ‘sustainable’ plant-based diets for children, whose possible nutritional deficiencies are not taken into account. This has important implications for the present article.

## Current dietary trends in the Western world and their long-term relationship to obesity and type 2 diabetes

**Figure 3.**
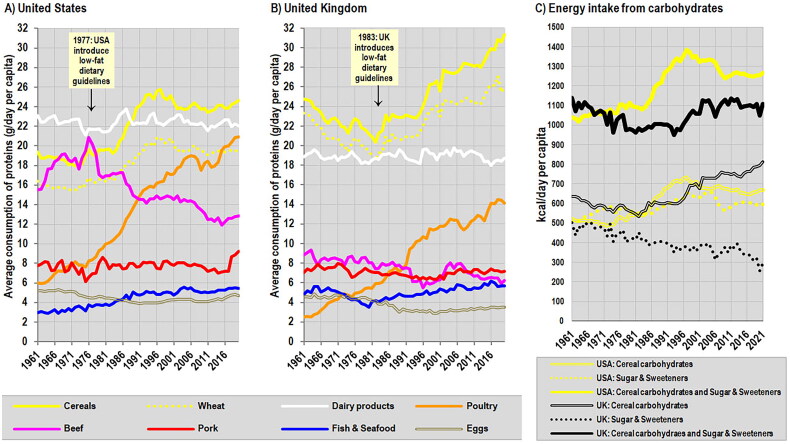
(A) Trends in protein consumption from main food items in the United States between 1961–2020. (B) Trends in protein consumption from main food items in the United Kingdom between 1961–2020. (C) Trends in energy intake from main carbohydrate sources in the United States and the United Kingdom between 1961–2021. *Source:* FAOSTAT: Food balances [14]. *Note:* FAOSTAT data for the period 1961–2013 are according to the older FAOSTAT methodology. FAOSTAT data on protein consumption between 2014–2020 (Figure 3A–B) are according to the new FAOSTAT methodology, but before the 2021 update and subsequent revision. FAOSTAT data on energy intake from carbohydrates between 2014–2021 (Figure 3C) are according to the newer FAOSTAT methodology, with revised and recalculated values. Energy intake from cereal carbohydrates is estimated by subtracting protein energy (4.1 kcal/g) and fat energy (9 kcal/g) from total energy.

Based on the positive experience with the FAOSTAT statistics and their applicability to the individual level, it would be possible to assess current nutritional trends in Western countries and predict the future occurrence of positive or negative health phenomena. This can be seen in Figure 3A–B, which clearly show the immediate impact of the low-fat dietary recommendations in the United States (1977) and the United Kingdom (1983). Given that cereals had been declared the basis of the human diet, they became the main source of protein and energy, whereas the consumption of red meat (beef, pork) and eggs decreased, and red meat started to be replaced by poultry. Evidently, the persistent efforts to link the increasing prevalence of type 2 diabetes in the United States to red meat consumption (cf. [46]) defy elementary logic. Instead, these trends are in perfect accordance with the higher glycemic load of the diet because refined cereals (and potatoes) have the highest glycemic and insulin indices out of all common foods [47]. This factor was recently highlighted even by the PURE study [48] and is consistently associated with type 2 diabetes in the meta-analyses of observational studies [30,31,49,50]. The list of recognized risk factors also includes sweeteners containing low-glycemic fructose (i.e. refined sugar or corn syrup), which negatively affects hepatic metabolism [51]. Although greater importance is usually attributed to sweetened beverages than to refined cereals and potatoes, these relationships may be country-specific because the consumption of sugar & sweeteners in Europe is mostly much lower than in the United States (Figure 3C).

Analyses of long-term dietary trends in the United States have also been conducted by other authors, who obviously use similar sources because there is a visible agreement with the FAOSTAT data [52–54]. Although the accuracy of the food supply statistics reaching as far back as the beginning of the twentieth century is not possible to verify, both the data by Lee et al. [53] and Wiegers et al. [54] indicate that carbohydrate consumption in the United States gradually decreased throughout the twentieth century, reached an all-time low around 1970, and then increased again. The present-day consumption of carbohydrates is probably similar to the early twentieth century, but total energy intake is ∼20% higher. At the same time, we can assume that energy demands are substantially lower due to generally lower work-related physical activity. In general, the American diet is characterized by historical tendencies toward an increasing proportion of highly processed foods with low nutritional value, mainly in the form of refined cereals, sweeteners, and vegetable oils. From this perspective, the current promotion of plant-based diets certainly does not seem to be an ideal solution. Furthermore, some highly processed forms of carbohydrate sources (potato fries, breakfast cereals) suffer from protein denaturation [55], which further exacerbates their low protein quality.

Bentley et al. [52] and Wiegers et al. [54] also attempted to correlate the long-term development of food supply with the prevalence of obesity and cardiometabolic diseases but focused mainly on sugar and sweeteners, without considering the role of other carbohydrate sources. Bentley et al. concluded that there was no apparent relationship between the increase in obesity rates and excess dietary sugar, and obesity could be explained by a delayed effect of cumulative sugar intake. The study by Wiegers et al. examined long-term changes in dietary protein, fat, carbohydrates, and added sugar but was unable to identify any specific positive correlation of these factors with selected health indicators (metabolic syndrome, obesity, type 2 diabetes), except for a general association of type 2 diabetes with increasing food intake.

**Figure 4.**
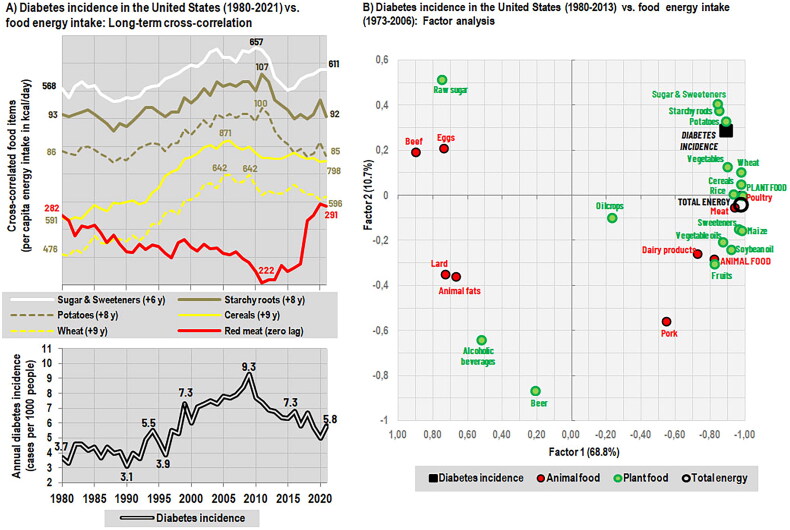
(A) Age-adjusted incidence of diabetes in adults (type 1 and 2) in the United States (1980–2021) plotted against average per capita energy intake (kcal/day) of selected food items. Data on food energy intake have been shifted forward in time, according to their strongest cross-correlation with diabetes incidence between 1965–2021. The underlying analysis includes 27 food items with an average per capita energy intake of ≥ 50 kcal/day between 1965–2021. Potatoes accounted for 92.6% of the total consumption of starchy roots in the years 1965–2021. Note that between 2014–2021, beef consumption is differently calculated and red meat consumption is ∼7–9% higher than it would have been in the preceding period. (B) Factor analysis of the age-adjusted incidence of diabetes in adults (type 1 and 2) in the United States (1980–2013) and 27 foods with an average per capita energy intake of ≥ 50 kcal/day (1973–2006). Data on food energy intake have been shifted seven years forward in time. *Sources:* FAOSTAT: Food balances [14]; Centers for Disease Control and Prevention, CDC.gov (obtained through personal communication). *Note:* FAOSTAT data for the period 1961–2013 are according to the older FAOSTAT methodology. FAOSTAT data for the period 2014–2021 are according to the newer FAOSTAT methodology, with revised and recalculated values. The differences in energy intake between 2010–2013 are the largest for beef (+21.0% according to the new methodology), soybeans (+19.4%), vegetables (+13.5%), and starchy roots (+10.7%).

In the present study, the ‘cross-correlation’ tool in Statistica 14 software was used to compare the trend line of diabetes incidence in the United States (1980–2021) with the trend lines of daily per capita energy intake from 27 staple foods in the FAOSTAT database (1965–2021, i.e. with a realistic causal lag up to 15 years). Although the diabetes statistics included both type 1 and type 2 diabetes, type 1 is a marginal form of diabetes in the United States, and the potential error would therefore be very small. In this way, it was possible to identify three food items that were by far the most associated with the development of diabetes incidence with a lag of 6–8 years: Sugar & sweeteners (*r* = 0.79, six-year lag), potatoes (*r* = 0.75, eight-year lag), and starchy roots (*r* = 0.74, eight-year lag). Wheat (*r* = 0.60, nine-year lag) and cereals as a whole (*r* = 0.57, nine-year lag) were also significantly correlated (*p* < 0.05), but less so than we would have expected based on their consumption rates and the glycemic index of refined grains (Figure 4A). Whether this result could have been influenced by the contemporary promotion of whole grains is difficult to say, as FAOSTAT does not distinguish this item. An alternative analysis working only with the older version of FAOSTAT (1965–2013) and diabetes incidence between 1980–2013 yielded even more convincing results, pointing to sugar & sweeteners (*r* = 0.89), starchy roots (*r* = 0.87), and potatoes (*r* = 0.82) (*p* < 0.05). The factor analysis in Figure 4B displays these relationships visually, although they are not identical, as this statistical method works with linear correlations.

Long-term time series of recent trends in diabetes incidence (≥ 20 years) from other countries are rare. The longest statistics of this kind are available from Canada [56], Denmark [57], Portugal [58], and the United Kingdom [59] but the data from Denmark (1996-2016) obviously suffered from methodological issues and did not produce any meaningful findings, and those from Portugal consist of three-year averages. Age-standardized incidence of type 1 and type 2 diabetes in Canada (calculated as the average of both sexes) concerns only the province of Saskatchewan and is complete in the period 1980–2003. A cross-correlation with the national-level data of food energy intake (1965–2003) shows almost perfect agreement with the trend line of soybean oil (*r* = 0.98, zero lag), which is primarily used for cooking and therefore represents culinary practices rather than dietary intake. Other significantly (*p* < 0.05) and strongly associated food items are cereals (*r* = 0.83, one-year lag), total energy (*r* = 0.78, two-year lag), and wheat (*r* = 0.78, three-year lag) (Supplementary Figures 1A-1B). A cross-correlation of type 2 diabetes incidence in the United Kingdom between 1991–2010 and food energy intake between 1976–2010 identifies cereals (*r* = 0.76, zero lag) as the strongest dietary predictor, followed distantly by wheat (*r* = 0.67, one-year lag), and total energy (*r* = 0.66, two-year lag) (*p* < 0.05). Although sugar & sweeteners do not reach significance because their consumption was gradually decreasing, their combination with cereal carbohydrates is particularly closely associated with long-term incidence rates (*r* = 0.90, zero lag) (Supplementary Figures 2A-2B). Interestingly, the incidence of type 2 diabetes has increased in parallel with increasing physical activity in UK adults [59], suggesting that physical activity is unlikely to be an important confounding factor.

As can be seen, apart from the anomalous cross-correlation of soybean oil in Canada (whose direct causal connection is questionable but may reflect kitchen preparation of obesogenic foods), the statistical comparisons from all three countries provide overall consistent results that are in line with current knowledge and point to high-glycemic carbohydrates and sweeteners as the main dietary triggers of type 2 diabetes. It is also meaningful that these relationships are potentiated by high energy intake and contribute to the development of obesity. At the same time, the compelling nature of these findings, which are based on incidence rather than prevalence and take advantage of long-term synchronicity (thereby greatly reducing the number of possible confounders), offers a promising methodological alternative to observational studies. However, this only applies if reliable long-term statistics are available, which is still complicated by changes in diagnostic methods and diagnostic thresholds. For example, the introduction of glycated hemoglobin (HbA1c) as a diagnostic tool, which began in the United States in 2010 and in Denmark in 2012, may have contributed to the subsequent decline in diabetes incidence. On the other hand, any underestimation of diabetes incidence would have led to a delayed increase in subsequent years, which happened in Denmark but not in the United States. Differences in the quality of diagnostics, as well as different rates of consumption and overly linear, indistinct trend lines, could potentially explain inter-country discrepancies in lag times.

In all three countries, we also observe that total energy intake from red meat (beef, mutton, pork) is significantly inversely related to diabetes incidence. In the United States, the cross-correlation reaches *r* = −0.47 between 1965–2013 (zero lag) and *r* = −0.43 between 1965–2021 (zero lag). In Canada (*r* = −0.64, six-year lag) and the United Kingdom (*r* = −0.50, seven-year lag) (*p* < 0.05), it is even stronger. This finding is in irreconcilable contradiction with the results of observational studies, which, in addition to dietary carbohydrates, link type 2 diabetes with the consumption of red meat [31,49,50]. However, these observational findings are, virtually without exception, strongly confounded by obesity and bad lifestyle habits, and lack a credible rationale.

To illustrate the chronic problems of observational methodology, we can mention a study by Etemadi et al. [60], who used a large cohort of the National Institutes of Health (NIH)-AARP Diet and Health Study in the United States. The authors associated self-reported red meat consumption with mortality from numerous chronic diseases, including stroke and type 2 diabetes, and the highest hazard ratio was observed between red meat and mortality from respiratory and liver diseases. Given that the most likely cause of the former is smoking and exposure to pollutants [61], and the latter is usually related to drinking alcohol [62], one can express serious doubts about the validity of these findings, despite the authors’ seemingly successful efforts to control for some of these lifestyle variables. In fact, one paper analyzing data from the NIH-AARP Diet and Health Study found a highly significant association between red meat consumption and accidental death in men [63], illustrating the difficulty of interpreting these relationships in any meaningful way.

Another case is the recent, widely publicized observational study by Gu et al. [46], which linked red meat to type 2 diabetes in a large sample of the Nurses’ Health Study and the Health Professionals Follow-up Study. The self-reported data on food consumption used by the authors were so implausible that they must have been artificially ‘calibrated’, not to mention that the most likely causal factors (cereals, potatoes, sugar, sweeteners) were not even included in the presented analyses and readers had to be content with a brief note that the results were adjusted for glycemic index and refined grain consumption. Interestingly, a similar team reported in their previous paper [64] that the documented positive relationship between animal protein and mortality was only evident in participants with at least one unhealthy lifestyle factor, confirming the strong confounding of this research by healthy user bias.

These examples are by no means rare and show that the dietary policy directed against the consumption of red meat (and animal foods in general) is based on very unreliable and biologically incompatible evidence influenced by hard-to-deny biases. The alleged link between red meat and type 2 diabetes still remains purely speculative [65], as meat is not a source of carbohydrates and has a zero glycemic index and a low insulin index [47]. Furthermore, red meat does not differ in this respect from other animal products. The fact that self-reported red meat consumption has recently been associated with high fasting glucose and insulin levels [66] is also inconsistent with the results of controlled clinical trials that failed to find such a relationship in healthy individuals [67,68].

## Optimizing protein recommendations for children

To further highlight the complexity of the actual situation, the FAOSTAT database can also be used to assess parallel changes in dietary protein quality. However, identifying the proteins most valuable for the physical growth of children is problematic because it is practically impossible to conduct a well-controlled, long-term study at the individual level, and this topic is thus still insufficiently researched [69]. There are also relatively limited data regarding the currently promoted DIAAS [Digestible indispensable amino acid score] of protein quality, which is based on the digestibility of each individual amino acid and replaced the PDCAAS [Protein digestibility-corrected amino acid score] in 2011 [70]. The most recent meta-analysis of available DIAAS scores [71], which assessed the amino acid composition of foods according to the new FAO/WHO/UNU standard 2007 [69], found that in the most reliable pig model, complete milk protein achieves the highest value of protein quality (136.0 on average), followed by pork (125.7), eggs (122), and beef (104.8). Plant protein sources – from sorghum (29) and wheat (43) to soy flour (105) – are clearly inferior in this regard. The combinations of cereals and common legumes, which are often recommended as a replacement for animal foods, never actually come close to the complete amino acid spectrum according to the older FAO/WHO 1985 standard [72] and only very rarely approach the DIAAS value of 100 according to the newer FAO/WHO/UNU 2007 standard. Moreover, these combinations look even less optimistic in practice due to the disproportionate volume and energy content of plant foods. For example, a 2:1 ratio of boiled peas and boiled rice would achieve a complete DIAAS of ca. 100 [71], but their nutritional composition [73] shows that to obtain 30 grams of complete protein from this combined dish, we would have to consume 693 grams (3785 kJ), which is quite incomparable to a lean roasted pork loin (74 g, 580 kJ).

**Figure 5.**
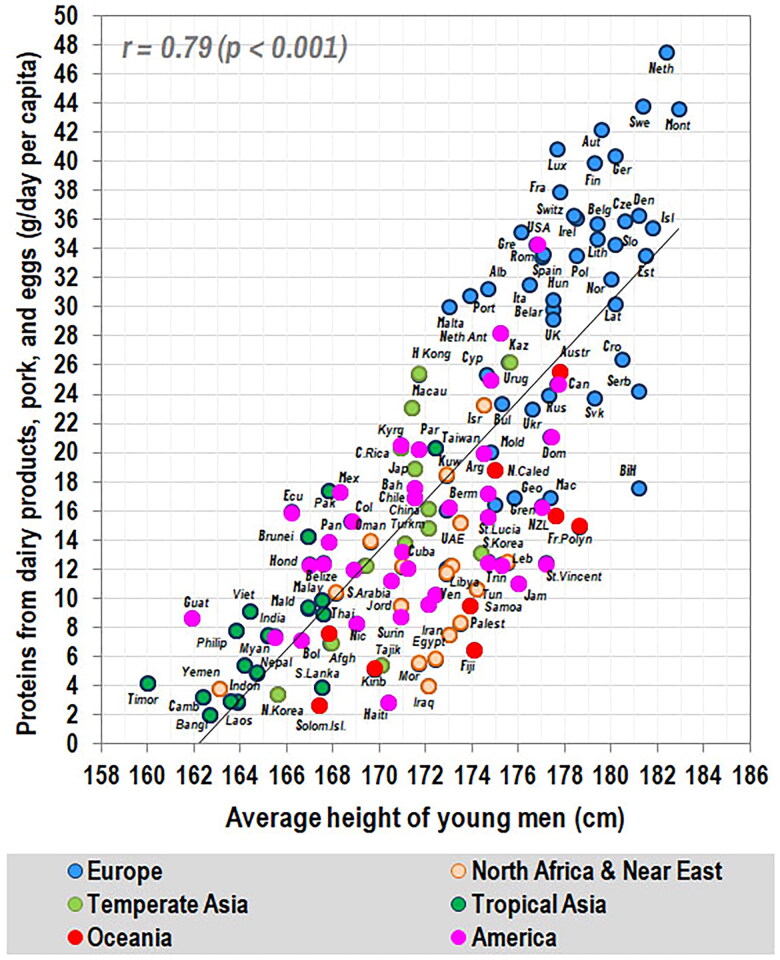
Relationship between male height in 136 populations and the mean consumption of proteins from dairy products, pork, and eggs (g/day per capita, FAOSTAT 1995–2013). *Source:* Grasgruber and Hrazdíra [74], with updated values for Finland, Hungary, The Netherlands, Romania, and Slovenia.

The problem of identifying key sources of protein in the human diet can also be viewed from the perspective of an ecological comparison, using average male heights in 136 world populations and the FAOSTAT data on daily per capita protein consumption [74]. In this case, the ecological approach has an invaluable advantage because the FAOSTAT statistics make it possible to trace the dependence between final height and long-term food consumption over the growth period, which is not possible in the real world. According to this comparison, dairy products, pork, and eggs are the main protein sources associated with height worldwide (Figure 5), with potatoes possibly having some slightly additive effect. The role of beef and fish & seafood is rather marginal and region-specific, and poultry correlates negatively with height in highly developed countries. Child undernutrition is consistently predicted by plant proteins from cereals and legumes.

**Figure 6.**
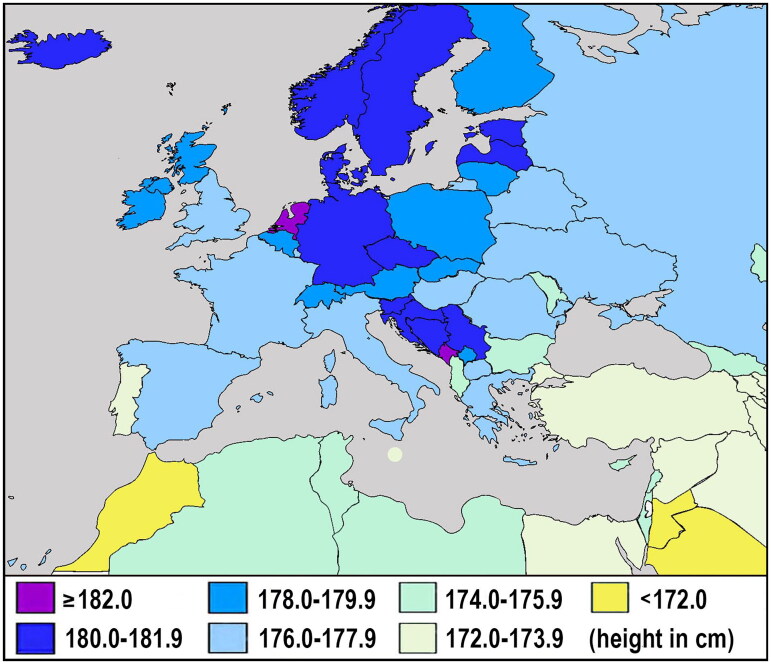
Average height of young men in Europe and adjacent regions (data from 2004 to 2018). As the influence of environmental factors begins to approach its maximum, current inter-country differences are increasingly dependent on inherited genetic predispositions. These are particularly pronounced in Scandinavia, the Baltic region, and the Western Balkans. *Source:* Grasgruber & Hrazdíra [74], with updated values for Finland, Hungary, the Netherlands, Romania, and Slovenia.

Given that with increasing economic wealth, the daily intake of proteins unavoidably reaches certain limits and we can also expect an increasing proportion of food waste, the distribution of male height in 44 European countries is best predicted by the ratio between the most consumed proteins of the best and the worst quality (dairy & pork/wheat) (*r* = 0.62, *p* < 0.001). The true effect of this ‘protein index’ at the country level is undoubtedly compromised by genetic factors (Figure 6) and is, therefore, much stronger in reality.

**Figure 7.**
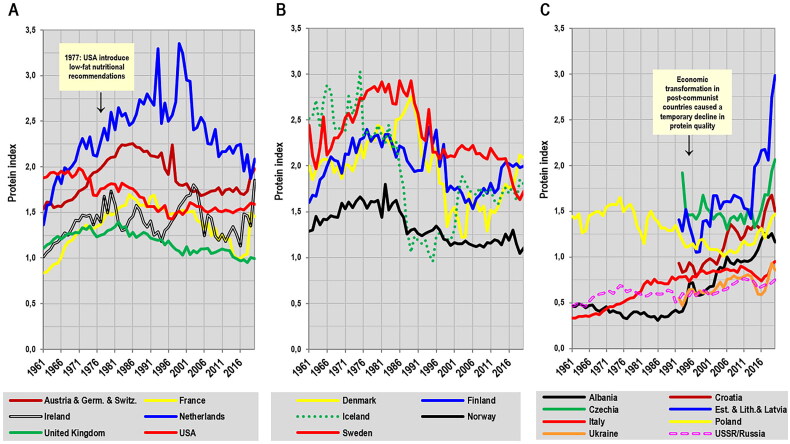
Historical changes in the protein index (the ratio between the annual consumption of proteins from dairy & pork/wheat, g/day per capita) between 1961–2020. (A) English-speaking and West European countries. (B) North European countries. (C) Post-communist countries and the Mediterranean region. *Source:* FAOSTAT: Food balances [14]. Data for the period 1961–2013 are according to the older FAOSTAT methodology. Data for the period 2014–2020 are according to the new FAOSTAT methodology but before the 2021 update and subsequent revision.

Remarkably, the values of the protein index have been declining in most affluent Western countries. The beginning of this process can be directly connected to the introduction of low-fat dietary recommendations in the late 1970s/early 1980s, but outside the Anglo-Saxon countries, it manifested with a certain delay (Figure 7A-B). Over the past decades, the drop of this indicator in Norway and the United Kingdom has been so significant that these countries have fallen below the level of Albania, where we observe a completely opposite trend, similar to other East and South European countries, which are still catching up with Western Europe nutritionally and economically (Figure 7C). The increase in protein quality in the Baltic states is particularly remarkable because they have taken over the baton of the best-fed nations of the contemporary world from the Netherlands.

Another noteworthy finding is the extraordinarily linear relationship between height and high-quality protein sources, with no signs of a plateau (see again Figure 5). This is contrary to the current recommendations of international organizations, according to which European children should be in a state of protein excess: The estimated daily protein intake in European children aged 2–9 years is ∼2.7 g/day [75], which is 2–3 times higher than the recommended intake of ∼0.7–1.0 g/day [76], assuming that these recommendations represent proteins with a complete amino acid spectrum. Therefore, at first glance, it seems clear that higher protein consumption should not bring any further benefits in terms of physical growth. However, it is important to realize that these recommendations are based on theoretical calculations derived from short-term experiments in adults and their validity for children is questionable. It is thus not too surprising that they have been challenged by more sophisticated methods showing that the protein requirements of children are markedly underestimated [77].

Although all these ecological results would not be considered a reliable basis for official nutritional recommendations, it is important to note that their validity has been repeatedly observed in the research practice of our university team. The key role of dairy as a predictor of physical growth was quite evident in a recently completed study that examined the relationships between height, body composition, and lifestyle in 2045 Czech high school students aged 18–22 years [78]. Dairy products were specifically chosen because they are easily identifiable dietary components and the accuracy of self-reported information could potentially be verified by correlation with height. This was especially true for boys, for whom the proportion of daily dairy consumers increased very linearly across height quintiles (*r* = 0.98, *p* = 0.004). Other food items were not monitored, but the important nutritional role of pork emerged in another anthropometric study in Bosnia and Herzegovina, where we were struck by the fact that Muslim high school students were routinely 2–3 cm shorter than their Croatian and Serbian peers living in the same regions or even in the same cities [79]. This unexpected result was explained when it turned out that the regional averages of body height correlated with the regional production of pork, which Muslims do not consume for religious reasons. This finding is even more remarkable when we consider that the average pork consumption in Bosnia and Herzegovina is among the lowest in Europe.

This practical experience shows that it would be possible to design observational studies whose results would meaningfully support each other (preferably using biochemical measurements) and thus produce credible nutritional information. Unfortunately, this cannot be said of most contemporary research. For example, a recent Adventist study of US youth aged 12–18 years [80] associated animal protein and meat consumption with the risk of obesity, but unlike our team’s study cited above, the authors were unable to document any correlation between self-reported food consumption and height. The absence of such a connection could be influenced by the faster maturation of obese children at the onset of puberty [81], but it is more likely that, similar to other observational studies of this type, the data used were inherently imprecise and very far from reality. The results of this study also contradict the inverse relationship between body height (quality animal nutrition) and obesity, which will be discussed in more detail later in this text.

## Decreasing protein quality in affluent Western countries leads to a reversal of the positive height trend

**Figure 8.**
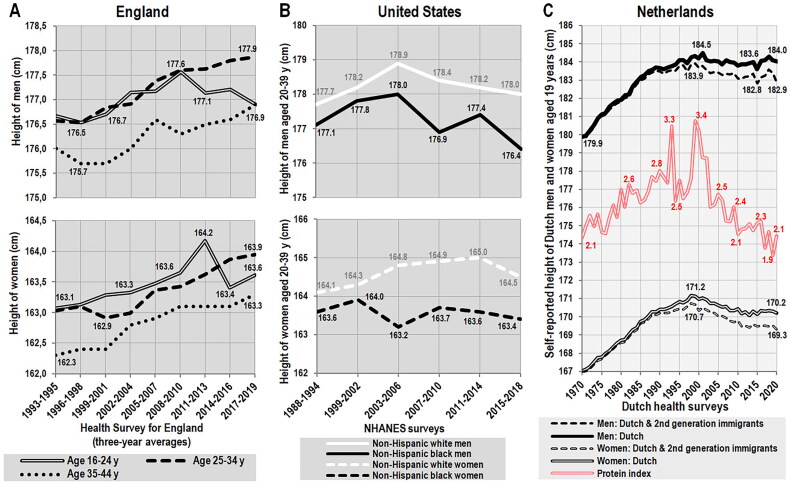
(A) Trend of measured body height in the United Kingdom between 1993–2019, according to the Health Survey for England 2021 [82]. (B) Trend of measured body height in the USA between 1988–2018, according to NHANES surveys [83]. Data for 1988–1994 represent a weighted average of the age groups 20–29 and 30–39 years, as they were combined during later surveys. (C) Trend of self-reported body height in the Netherlands between 1970–2020, according to annual health surveys [92].

In accordance with the decline in protein quality observed in the FAOSTAT statistics, the most recent results of nationwide health surveys performed in the United Kingdom [82] (Figure 8A) and the United States [83] (Figure 8B) document a gradual decline in body height in the youngest generations. Expectedly, it is more pronounced and faster in males, who naturally have higher requirements for nutrients than females during physical growth [84]. The exact beginning of this negative development cannot be determined because age groups in these health surveys have a very wide range. Still, in the United States, this trend appears to have fully hit the generation born in the late 1970s and in England, it started somewhat later, in accordance with the fact that a visible change in dietary protein quality did not occur until the early 1990s.

Given that the UK figures come from the annual Health Survey for England, which does not normally separate out ethnic minorities, it could be argued that the observed decrease in height is also due to an influx of non-white immigrants (mainly from India, Pakistan and sub-Saharan Africa), whose share in the United Kingdom progressively increased from 7.9% in 2001 to 12.8% in 2011 and 17.0% in 2021/2022 [85]. However, this survey only includes people living in private households, which means that it excludes students and temporary workers who make up the majority of England’s immigrant population. Naturalized non-EU immigrants with a UK passport made up only 5.5% of UK citizens in 2021 and the UK immigrant population is slightly under-represented in the age category of 16–25 years, being disproportionately concentrated in the working age group of 26–64 years [86]. Thus, even if these naturalized immigrants had a negative impact on average height, we would expect this to be particularly pronounced among older age cohorts – which apparently is not the case.

In the United States, the NHANES surveys offer data of suboptimal quality, suffering from an overly wide age range and relatively small sample sizes. The latter factor can explain the lower temporal stability of height trends in non-Hispanic US blacks. Because the number of non-Hispanic US whites is around 200 million and tends to decrease [87], and the black immigrant population grew rather modestly by 2.2 million between 2000 and 2019 [88] (5.4% of the total US black population in the 2020 census [87]), we can again rule out immigration as a significant driver of the documented changes, as it is easy to calculate that they would require tens of millions of short-statured immigrants.

A visible reversal of the height trend can be observed even in the Netherlands (Figure 8C), the case of which is very specific because this country has long resisted dietary policies targeting saturated fat and during the 1990s, the quality of proteins in the Dutch diet reached the highest level in the world (Figure 7A). Consequently, the Dutch population officially became the tallest in the world [89]. Available health statistics also show that the prevalence of obesity, type 2 diabetes, raised blood glucose, and cardiovascular disease in the Dutch population was one of the lowest in Europe and among all developed countries in general [7,17]. It is, therefore, somewhat surprising that the Dutch government has recently adopted quite radical plant-based dietary guidelines [90,91]. As a result, between 1999–2020, the per capita consumption of proteins from pork decreased by ∼48%, whereas the consumption of proteins from cereals increased by ∼57% [14]. The Dutch diet therefore follows the same development as in the United States and the United Kingdom (Supplementary Figures 3A-3C), and the consequences are also identical: The Fifth Dutch Growth Study 2009 [89] documented a stagnation of height in young men and women, and self-reported data from annual health surveys [92] indicate a slow height decline in 19-year-olds starting around 2002–2003 (Figure 8C). This downward tendency is very clear in women (–1.0 cm in ethnic Dutch women and −1.4 cm in a combined sample including 2^nd^ generation immigrants), but less so in men, which can most likely be attributed to the well-known fact that men frequently overestimate their height [93]. The association of the height trend with the protein index between 1970–2020 is evident, but according to the cross-correlation, the trend lines do not have exactly the same shape and reach significant similarity (*p* < 0.05) only in the combined sample (with a lag of 0–1 year for men and 0–3 years for women), showing the immediate impact of protein quality on young individuals with unfinished growth. Remarkably, the decrease in height occurred despite the fact that the values of the protein index were still very high, and since their decline further accelerated, we can expect that the negative height trend will continue in the generation born during the 2010s.

The phenomenon of decreasing stature in these three Western countries has particularly serious implications for the United Kingdom and the United States, as they last experienced a similar crisis in the mid-nineteenth century. It was apparent even in some countries of continental Europe and was connected with societal and economic difficulties accompanying the onset of the Industrial Revolution [94,95]. Only after overcoming these problems was the height gain continuous and often staggering: For example, the mean stature of Austrian men increased from ∼161 cm around 1790 [96] to ∼179 cm during the last decade [97,98].

Although the positive height trend has been driven by rising GDP (gross domestic product) per capita, it is important to realize that economic, social, and demographic indicators act only as mediators of the real biological determinants of physical growth, which are genetic factors (particularly evident in the Western Balkans) and nutrition. Economic historians recognize that the utilization of nutrients can be negatively affected by the occurrence of environmental stressors, especially infectious diseases (represented by child mortality statistics), which exhaust the growth potential of the child’s organism [99]. Also noteworthy is the independent position of total fertility in the regression analyses of height, as it influences the distribution of resources within families and reflects more subtle aspects of childcare, such as the length of breastfeeding [74]. The most informative socio-economic variable with the greatest predictive power (*r* = 0.83, *p* < 0.001 in 96 countries) is the inequality-adjusted Human Development Index (IHDI), which combines GDP per capita, life expectancy, and the level of education [74]. As a result, height is a very sensitive measure of improving living conditions, even independently of economic development, and its sudden decrease (after ca. 150 years of continuous growth) is a warning sign that the health in Western countries is again deteriorating. This is, of course, clearly demonstrated by the statistics in Figures 1A–C and 2.

**Figure 9.**
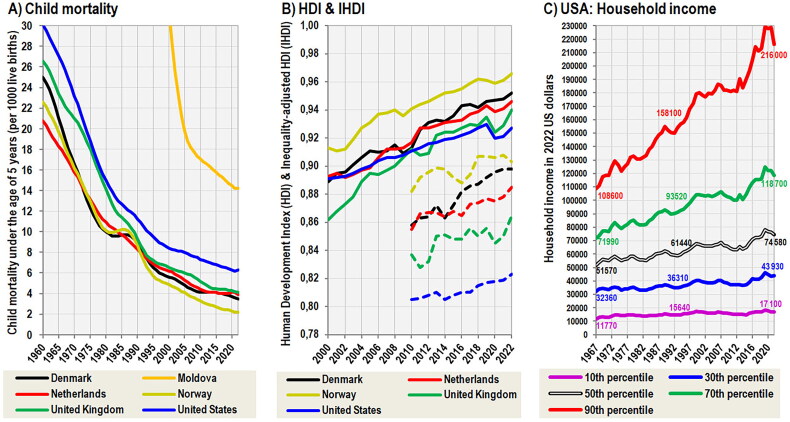
(A) Child mortality under the age of 5 years between 1960–2022, according to the World Bank [100]. (B) Human Development Index (HDI) and Inequality-adjusted Human Development Index (IHDI) between 2000–2022, according to the Human Development Reports [101]. IHDI values are indicated by a dashed line. Note the temporary decline in these indicators associated with the economic crisis in 2008 and the COVID epidemic in 2020. (C) Household income dispersion in the United States between 1967–2022, according to the US Census Bureau [102].

Crucial to the current analysis is the fact that the main indicators of economic wealth and health care (GDP per capita, health expenditure per capita, child mortality, IHDI) have been steadily improving in highly developed Western countries in recent decades (Figure 9A–B) and total fertility has stabilized [100,101]. Although the value of the Gini index (social inequality) in the United Kingdom and the United States visibly increased to ∼40 during the 1990s [100], median household income increased as well [102] and the greater wealth gap is due to the fact that the richest social strata became disproportionately richer (Figure 9C). Therefore, the negative effect of socio-economic factors and environmental stressors is already irrelevant or at best very marginal, and after excluding the role of immigration, the principal denominator of the current decline in height must be sought in the deterioration of dietary protein quality. This can be further demonstrated by the example of regular measurements of recruits in North European (Denmark, Finland, Norway, Sweden) and German-speaking (Austria, Germany) countries, which are characterized by high social equality (Gini index 32 >) and a highly developed social and health system.

**Figure 10.**
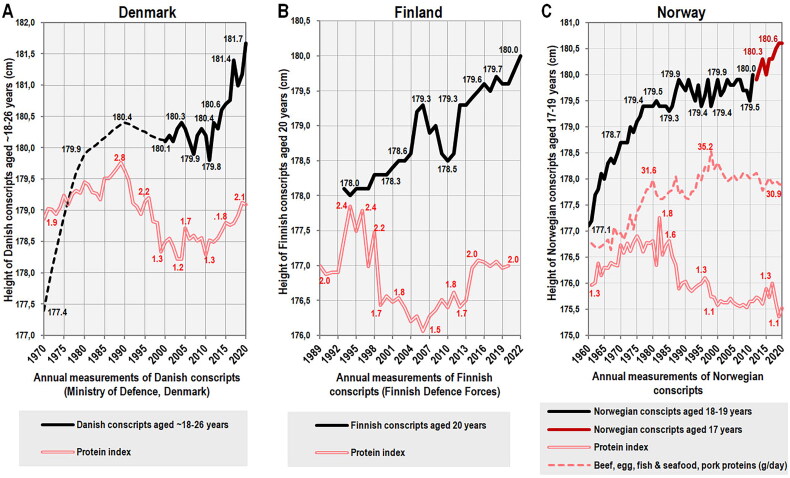
**(** A) Trend of measured body height in Danish conscripts (all men liable for military service) between 1970–2020, according to the Ministry of Defence, Denmark [103]. Data on body height prior to 2000 are incomplete and marked with a dashed line. (B) Trend of measured body height in Finnish conscripts serving in the military between 1993–2022, according to the Finnish Defence Forces [104]. (C) Trend of body height in Norwegian conscripts (all men liable for military service) between 1960–2011 (measured height in 18–19-year-olds) and 2012–2021 (self-reported height in 17-year-olds), according to the Statistics Norway [105] and the Statistical Yearbook [106].

In Denmark [103], the height of conscripts aged ∼18–26 years (with 18-year-olds making up ∼60% of the total) experienced a slight but long-term decline from the 1990s to 2011 (Figure 10A), which remarkably agrees with the concurrent drop in dietary protein quality: Between 1973–2010, cereal protein consumption steadily increased by ∼49%, whereas pork protein consumption dramatically decreased by ∼60% in the late 1990s (Supplementary Figure 4A). As a result, the protein index started to decrease in 1990 and reached the lowest values in documented history in 2002–2003. The rapid restart of the height trend after 2011 again corresponds very well with the reversal of the downward development of the protein index, which was caused by the growing consumption of dairy products. Although the trend lines of height and protein index between 2000–2020 are not similar in terms of the cross-correlation analysis (*p* > 0.05), the relationship is quite strong when expressed by simple Pearson linear correlations (*r* = 0.77, *p* < 0.001 at a one-year lag).

A very similar development of the height trend can be observed in 20-year-old conscripts in Finland [104]. Here, the protein index temporarily decreased during the 2000s due to the increasing share of cereals in the diet and reached an all-time low in 2006. The decline in the protein index was followed, with a four-year delay, by a short-term reversal of the positive height trend (Figure 10B). After the increase in dairy consumption during the 2010s, the values of the protein index also increased and the positive development of height continued (Supplementary Figure 4B). Although there is again no long-term positive cross-correlation between the protein index and height, Pearson linear correlations identify their significant association (*p* < 0.05) after the profound decline in protein quality between 2002–2020, with a peak of *r* = 0.66 (*p* = 0.002) at a lag of three years. It should be added, however, that the number of measured conscripts decreased from >30,000 before 2003 to less than 10,000 in 2006–2009, and it is possible that the situation in this period is somewhat distorted.

Historical records from Norway [105,106] demonstrate a parallel development between height and the protein index in 18–19-year-old conscripts between 1961–1985, as shown by a strong linear correlation during this period (*r* = 0.69, *p* < 0.001). According to the cross-correlation, significant similarities (*p* < 0.05) can be found with lags of 0–7 years, with a peak at a lag of five years (*r* = 0.55). After 1985, dietary protein quality started to decline, mainly due to a sudden drop in the consumption of dairy products, which were replaced by cereals (Supplementary Figure 4C). However, unlike other North European countries, height did not decrease and began to stagnate from 1988 onwards (Figure 10C). Since 2012, only self-reported heights from 17-year-olds have been collected and although the most recent averages slightly increased, no conclusions can be drawn from such short-term and unreliable data, as the trend was exactly the opposite for girls – from 167.2 cm in 2016 to 166.8 cm in 2021.

**Figure 11.**
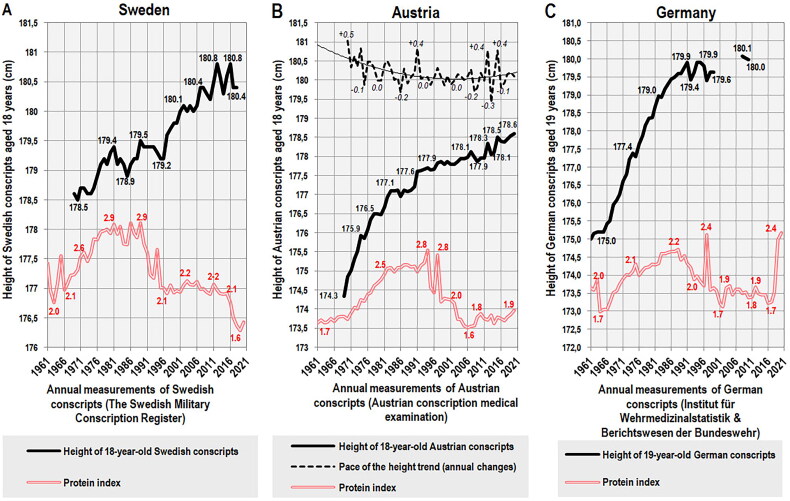
(A) Trend of measured body height in Swedish conscripts between 1969–2018, according to the Swedish Military Conscription Register [107]. (B) Trend of measured body height in Austrian conscripts between 1969–2020, according to the Austrian conscription medical examination [98]. (C) Trend of measured body height in German conscripts between 1961–1999 and 2008–2010, according to the Institut für Wehrmedizinalstatistik and Berichtswesen der Bundeswehr [109]. the data for the period 1961–1991 concern the former Western Germany. The data for the periods 1992–1999 and 2008–2010 concern the reunified Germany.

In Sweden, the height of 18-year-old conscripts [107] closely followed the protein index between 1969–1989 (*r* = 0.88, *p* < 0.001) and this is also evident from the cross-correlation, which identifies significant similarities at lags of 0–10 years, with a peak at zero lag (*r* = 0.69). After this period, we can observe a marked decline in protein quality, which was – similar to Finland – caused by the rising consumption of cereals (Supplementary Figure 5A). Still, this development seems to have only a slight and temporary negative effect on height because the protein index of the Swedish diet remained one of the highest in Europe, at the level of the Netherlands (2.1–2.2). Stagnation is only noticeable in the years 2012–2017, which suggests that at this nutritional status and the current level of socio-economic factors, the growth potential has already been exhausted (Figure 11A). Nevertheless, the interpretation of this situation is complicated by the high proportion of non-European immigrants: Whereas 18–19-year-old Swedes of Nordic origin from the Göteborg area reached 181.4 cm in 2008–2009 (∼1 cm above the contemporary height of conscripts), their peers of non-Nordic origin (13.6% of the sample) were only 177.7 cm tall [108]. It is possible that the dietary habits of Muslim immigrants (the prohibition on pork) contributed to the new downward tendency of protein quality following the 2015 migration crisis. In any case, as long as the level of nutrition remained high, even mass non-European immigration was unable to reverse the height trend. The current outlook, however, is far less optimistic.

In Austria, the height of 18-year-old conscripts [98] initially increased in parallel with the protein index between 1969–1994 (*r* = 0.96, *p* < 0.001) and a significant cross-correlation can be found at lags of 0–6 years (with a peak of *r* = 0.82 at zero lag). After this period, protein quality began to deteriorate as pork began to be replaced by cereals (Supplementary Figure 5B) and dairy protein consumption also declined significantly after 2003. As a result, the protein index reached its historical minimum in 2006. This dramatic decline in protein quality was similar to that in Finland, but the stature of the Austrian conscripts was shorter. As a result, the height trend did not reverse but more or less stopped for about a decade and a half (1998–2011). In 2009, the values of the protein index began to rise again due to the higher consumption of dairy proteins, and this was almost immediately reflected in the new upward trend of height starting in 2012 (Figure 11B).

The height of 19-year-old German conscripts [109] was also tightly tied with the protein index between 1961–1991 (*r* = 0.91, *p* < 0.001), with a significant cross-correlation at lags of 0–5 years (with a peak of *r* = 0.88 at zero lag). After this period, we observe a substantial deceleration to stagnation of the height trend. As the height averages for the period 1961–1991 refer only to the former West Germany and the height of East German conscripts in 1992 was 1 cm lower, this development could be explained by the inclusion of shorter East German recruits (Figure 11C). However, the height of conscripts in the former East Germany quickly approached the West German average (from 178.4 cm in 1992 to 179.8 cm in 2009) and the observed halt in the height trend is therefore real. The role of immigration is also very unlikely: Although immigrants or their 2^nd^ generation descendants made up 24.3% of the German population in 2022 [110], their naturalization was much more limited (3.51 million people between 1992–2010, i.e. ∼4.4% of the German population in 2011) [111]. In contrast, the time course of the long-term stagnation again agrees very well with the decline in protein quality, which started during the process of reunification in the early 1990s (Supplementary Figure 5C) and was rather mild. The rapid improvement in the protein index in 2019–2020 was due to a significant increase in the consumption of dairy products and should have a visibly positive impact on the youngest generation of young Germans.

## Implications for child nutrition and childhood obesity management

Taken together, this overview of long-term anthropometric data from nine countries shows that changes in protein quality (protein index) are very rapidly followed by changes in the height trend. Particularly compelling are the immediate responses of body height to fluctuations in the protein index in countries such as Austria, Denmark, Finland, and the Netherlands. Even more remarkable is the fact that this indicator is based on just three staple foods (dairy, pork, wheat), which points to their fundamental role in children’s nutrition. At the same time, there is no close long-term synchronicity between height and the protein index; rather, we can talk of a certain minimum value of the protein index that guarantees a continuous increase in body height. This value is undoubtedly the result of genetic and socio-demographic factors, and is currently around 2.0 in northern and central European countries. Its importance increases the more the given population approaches its genetic limits, as evidenced by the example of the Netherlands.

On the other hand, the protein index is certainly not the only dietary predictor of height and it is possible that the negative impact of nutrition may be compensated by the exceptional quality of the social environment and health care. This can be illustrated by the example of Norway, where the stature of conscripts has only stagnated for 24 years, despite continuing tendencies toward deteriorating protein quality. There may be at least two reasons explaining this paradox: First, Norway has had the highest inequality-adjusted Human Development Index in the world in recent decades [74]. Second, the Norwegian diet is disproportionately based on fish and seafood (∼15% of total protein consumption between 1980–2010) [14], which can provide proteins of decent quality. In fact, the height trend in Norway between 1961–2011 shows an almost perfect linear correlation (*r* = 0.89, *p* < 0.001) with the combined consumption of four high-quality, non-dairy protein sources (beef, eggs, fish & seafood, pork), which is also confirmed by the cross-correlation (*r* = 0.93, *p* < 0.05 at zero lag) (Figure 8C).

In light of these real-life observations, current protein standards for children are difficult to defend because even a seemingly excessive protein intake of 2–3 times the recommended amount – which is typical of children in affluent European countries – is unable to avert negative developments associated with contemporary dietary changes. This conclusion is entirely consistent with the ecological trend line in Figure 4 and supports the criticism of current recommendations recently raised by Elango et al. [77] based on the results of the IAAO [Indicator Amino Acid Oxidation] method. Because the essence of these dietary changes lies in replacing red meat and eggs with cereals and poultry, their inevitable consequence is an increasing glycemic load of the diet and it is very likely that the decline in body height in European countries will have the same consequences as in the United States, i.e. an increasing incidence of obesity and cardiometabolic diseases.

This assumption can be supported not only by the analyses of diabetes incidence, but it can be even better understood in the context of the results of the above-mentioned study of Czech high school students [78]. Its results showed that, in line with other research from developed countries [112,113], the percentage of body fat decreased with increasing social status (represented by parental education) and up to 60% of the theoretical variability in % body fat in linear mixed-effect models could be explained by the self-reported level of physical activity. However, a notable finding was the inverse relationship between height and % body fat (BMI), which was independent of physical activity. For example, young men in the first quintile of body height were on average 170.6 cm tall with 17.8% body fat, those in the third quintile were 180.1 cm tall with 15.7% body fat, and those in the fifth quintile reached 189.6 cm with 14.6% body fat.

**Figure 12.**
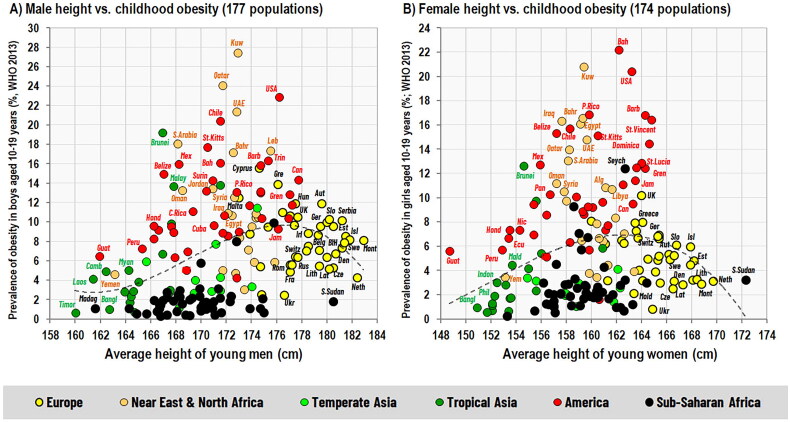
Relationship between height and childhood obesity in the global context (177 populations for males, 174 populations for females). Data on body height come from Grasgruber and Hrazdíra [74] and were supplemented by updated values and unpublished information on Sub-Saharan Africa. All these data are based on studies finished between 2004–2018 and were adapted to the time span of the environmental statistics examined (1995–2013). Data on obesity rates in children aged 10–19 years were taken from the Global Health Observatory [115] and are defined as % of children and adolescents with BMI two standard deviations above the median. The graphs do not include Oceania, due to the existence of a highly specific fat-free mass/fat-mass relationship in Oceanian populations, resulting in many outliers.

This phenomenon has been documented in many other countries [114] and data on childhood obesity rates from the Global Health Observatory [115] vividly illustrate that it is actually curvilinear in the global context: inverse in tall, well-nourished populations and positive in short, poorly-nourished populations (Figure 12A–B). Although its causes are still debated, the most likely explanation is the way of eating during growth, as this relationship gradually weakens with age in the adult population [116] and it can also change from positive to negative within a single country when nutrition improves, as documented in Switzerland over the last 150 years [117]. In the United States, on the contrary, the negative association between height and BMI disappeared in men and was significantly attenuated in women [118].

The polarity between height and obesity in growing children is completely logical because a diet based on high-quality animal proteins is not only a prerequisite for a low glycemic load but also guarantees the most efficient use of nutrients for physical growth. This is consistent with current scientific evidence showing that that high-protein diets may contribute to higher adiposity in preschool children, but have a favorable effect on the prevention of obesity in school-age children and adolescents, in whom they also promote the formation of lean body mass [76,119]. In contrast, a deficiency of essential amino acids leads to impaired protein synthesis, with excess amino acids being oxidized [120] and used for energy or stored as subcutaneous fat. The effect of an energetically adequate but nutritionally suboptimal diet can be illustrated by dietary patterns in Muslim countries of North Africa and the Near East, which are characterized by an extremely high prevalence of childhood obesity. The FAOSTAT statistics show that total energy intake and total energy intake adjusted for adult height are very similar to wealthy European countries, but both the proportion of animal protein and the average protein quality are substantially lower because the main sources of dietary protein are cereals (wheat) and poultry. In comparison, the consumption of high-quality dairy products is moderate at best, and the consumption of red meat is minimal because pork is forbidden for religious reasons. It is hard to ignore that this eating style closely resembles contemporary dietary trends in Western countries, which are accompanied by rising rates of obesity and type 2 diabetes.

In light of these biological rules, it is not difficult to understand why current recipes for preventing childhood obesity, still influenced by the stigmatization of animal foods, do not produce the desired results [121]. Usually, they contain stereotypical formulas encouraging the consumption of fruits and vegetables, without taking into account that these food components are marginal and cannot fundamentally influence the character of children’s diets. Although a low-glycemic, bulky plant-based diet also has the potential to reduce body weight [122], it is important to note that this strategy de facto replicates the dietary style of developing countries in Figure 12A–B, which will lead to nutritional deficiencies in the long term.

## Nutrition and phenotypic IQ

**Figure 13.**
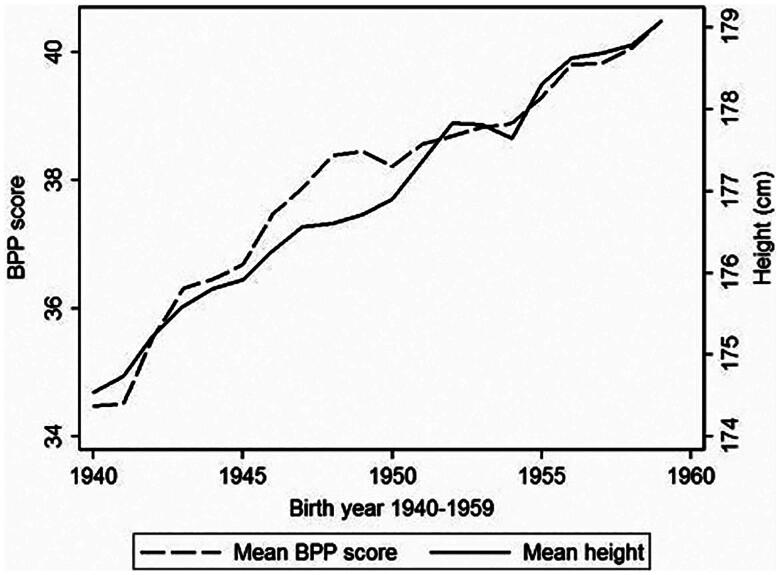
Average height (cm) and average intelligence score (Børge-Priens-Prøve score, BPP) across different years of birth in Danish conscripts (1940–1959). The Børge-Priens-Prøve (Børge-Prien’s test) is a test battery of four subtests, which was specially developed for the Danish Armed Forces after World War II and has remained unchanged to this day. *Source:* Christensen et al. [127] (with permission).

Another serious issue related to child nutrition concerns intellectual development. The average population IQ in Europe has been rising since the beginning of the Industrial Revolution, which has come to be known as the ‘Flynn effect’. Although the discoverer of this trend doubted the causal connection between IQ and improving nutrition, due to inconsistent gains in height and IQ across different social groups [123], the strong influence of diet on intellectual development is well documented. Undernutrition in developing countries is chronically associated with cognitive impairment [124] and there exists a close long-term correlation between IQ and height [125,126]. This can be graphically demonstrated by the example of Scandinavian conscripts [127], whose performance on intelligence tests increased in parallel with increasing stature (Figure 13). Nutritional factors are particularly important in early childhood when the brain experiences accelerated growth, and depend both on partial nutrients (e.g. iodine, iron) [128,129] and on protein quality that directly controls brain development [130,131]. A recent intervention study conducted on Kenyan children specifically highlighted iron, folate, and total energy as the strongest correlates of better cognitive performance, while zinc and vitamins B2 and B12 had significant predictive power only in certain special tests [132]. An important ‘hidden’ factor can also be the nutrition of the mother during pregnancy [133]. Consequently, an increase in phenotypic IQ caused by good nutrition can potentially mask the drop in genotypic IQ because the fertility rates of the lower social classes are disproportionately higher.

Based on these observations, we could reasonably expect that the decreasing quality of nutrition in many developed countries will be associated with a decrease in population IQ. Remarkably, this phenomenon does occur and is widely discussed. Actual data from several European countries indicate that phenotypic IQ has started to decrease and the Flynn effect has, therefore, reversed [134]. The same development was recently documented in the United States [135]. At first glance, this negative trend could be ascribed to the influx of immigrants, but a drop of ca. 2.5 points in 15 years was found even in ethnic Norwegian conscripts, not to mention that this decrease also occurred in conscripts coming from the same families, which points to some environmental factor [136].

**Figure 14.**
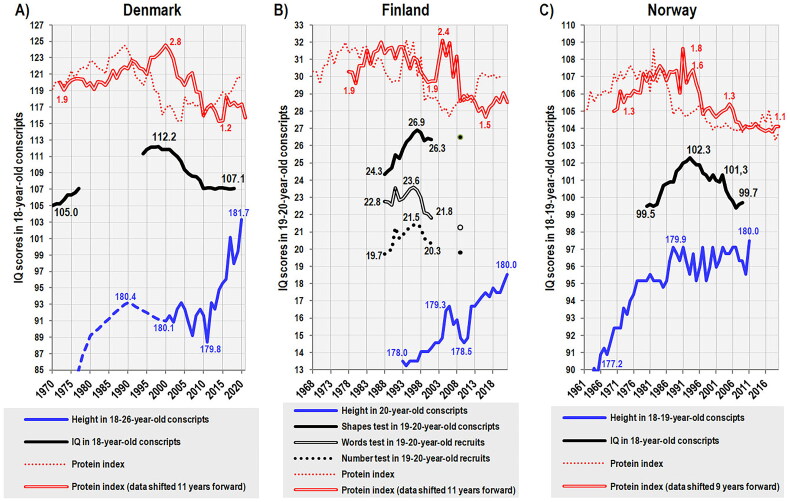
(A) Average results of IQ testing in Danish conscripts according to Hegelund et al. [137] projected against the protein index and the trend of body height. The IQ scores are derived from the Børge-Prien’s test (Børge-Priens-Prøve). The birth cohort of 1940 was chosen as the baseline (IQ score 100). Data on body height prior to 2000 are incomplete and marked with a dashed line. (B) Average results of IQ testing in Finnish conscripts according to Dutton and Lynn [138], projected against the protein index and the trend of body height. The IQ test of the Finnish Army *(Peruskoe)* consists of three subtests. (C) Average results of IQ testing in Norwegian conscripts according to Bratsberg and Rogeberg [136], projected against the protein index and the trend of body height.

The best-quality data for an ecological analysis of the relationship between nutrition, height, and IQ are available from Denmark and refer to military recruits born between 1952–1959 and 1976–2000 (corresponding to 18-year-olds tested between 1970–1977 and 1994–2018) [137]. Their performances in entrance intelligence tests started to decline in the 1981 birth cohort (18-year-olds in 1999), but this drop stabilized in the birth cohorts 1992–2000 (18-year-olds in 2010–2018). Using cross-correlation, we can observe that the values of the protein index show a significant association (*p* < 0.05) with the IQ of 18-year-olds when they are shifted forward by 4–17 years. The strongest correlation (*r* = 0.72) can be found at a shift of 11 years, i.e. when the examined conscripts were seven years old (Figure 14A).

In Finland, three different IQ tests of 19–20-year-old recruits are available for the period 1988–2001 and 2009 [138]. They show a slight temporary decline in 1992 and then a continuous regression between 1997–2009. Although cross-correlation shows that the Words test and the protein index are mutually synchronous (*r* = 0.68, *p* < 0.05), the two remaining tests do not reach significance and this also applies to the annual average of all three tests (not shown). However, given the shortness of this time series, the lack of statistical significance would be easy to understand. When the protein index is shifted 11 years forward (similar to Denmark), we can see that the decline in IQ test scores could potentially correspond to a temporary drop in protein quality during the late 1980s (Figure 14B).

In Norway, there are IQ data on the birth cohorts 1962–1991, corresponding to 18-year-olds between 1980–2009 [136]. When the IQ of 18-year-olds is projected against the protein index, cross-correlation identifies a significant association (*p* < 0.05) when the protein index is shifted 7–10 years forward. The strongest correlation can be found at a shift of nine years (*r* = 0.51), which is equivalent to an age of nine years (Figure 14C). Thus, even in Norway, we can document a temporal relationship between population IQ and protein quality, which peaks during a pre-pubertal age. Unlike Denmark, however, the decline in IQ was not accompanied by a decline in body height.

These limited statistics from three North European countries, only two of which are significantly associated with nutritional quality, cannot of course be taken as conclusive. Nevertheless, the delayed synchronicity of IQ values with the protein index in Denmark and Norway is definitely intriguing. It can be interpreted to mean that the temporal relationship between population IQ and nutrition is fundamentally different than that between population height and nutrition, and despite the long-term positive relationship between IQ and height, these two variables evolve differently in environments of declining protein quality. More specifically, the decline in height precedes the decline in IQ by roughly one decade, which could be explained by the fact that nutrition permanently affects children’s intelligence only until pre-adolescence, whereas height is acutely affected in maturing adolescents. Future trends in Denmark could be crucial to strengthen the causality of these findings, as the decline in phenotypic IQ stopped after the stabilization of dietary protein quality and a reversal of this negative development can be expected, similar to the case of body height.

Still, even if this development is confirmed, several problems also arise that require explanation. Above all, it cannot be overlooked that the relationship between protein quality, height, and IQ is not entirely consistent. In particular, it is not clear why changes in protein quality would have a greater effect on intelligence than on height in Norwegian recruits. Assuming that the level of education, social environment, and environmental stimuli in developed countries continue to improve, intelligence should be affected less than height. In addition, the drop in IQ scores in Denmark was also disproportionately greater compared to the slight downward trend in height. A speculative answer could be that some of the missing nutrients are more important for brain development than for physical growth. In this regard, meat appears to have more pronounced effects on IQ, whereas dairy products primarily promote linear growth [139], but both are superior to a plant-based diet [132]. Alternatively, nutrition worked in tandem with some other environmental factor(s).

Definitive illumination of these problems would require specialized research that would carefully consider the role of immigration, dysgenic genetic selection, the development of IQ by social status, and all the other environmental variables that theoretically come into play. For example, the standard deviation of the mean population IQ in Denmark was diminishing throughout the twentieth century, and the combination of height, family size, and especially education explained 99.6% of the variability in IQ values during this time [137]. In any case, the causality between IQ and nutrition has a meaningful biological basis, and underestimating the negative impact of current nutritional changes could have serious consequences for future civilization potential.

## Conclusion

The aim of the present article was to draw attention to the radical nutritional changes taking place in developed Western countries. These changes can be monitored through food statistics in the FAOSTAT database and consist of replacing red meat and eggs with cereals and poultry, worsening the average quality of dietary proteins, and increasing the proportion of carbohydrates consumed. They are closely accompanied by a decrease in body height (unprecedented since the end of the nineteenth century), an increase in the incidence of obesity and cardiometabolic diseases, and a decrease in phenotypic IQ (again unprecedented in the modern industrial age). The causality of these relationships has a meaningful biological basis since the decline in protein quality has a negative effect on physical growth and brain development, and a higher glycemic load leads to the development of many related health problems.

Because the decline in height occurs independently of immigration, and environmental stressors that once negatively affected children’s growth are no longer relevant in modern Western societies, no other known trigger for this negative trend can realistically be considered, apart from deteriorating nutrition. The increase in obesity and cardiometabolic diseases may have other causes besides diet, such as a decrease in physical activity, but the strong association between diabetes incidence and the consumption of high-glycemic carbohydrates and sweeteners in the FAOSTAT database is quite convincing and agrees with the evidence collected from observational studies. Furthermore, the prevalence of obesity in developed countries is inversely related to body height, and decreasing height (decreasing protein quality) is therefore interconnected with rising obesity rates. Declining population IQ may also be the result of various environmental and genetic (dysgenic) factors that cannot be adequately addressed in this commentary, and the data presented from three North European countries are too limited. Nevertheless, the strikingly synchronous relationship between protein quality and IQ of recruits in Denmark and Norway is remarkable and worthy of serious attention.

The most compelling implication of these observations is the fact that current recommendations for child nutrition should be radically reevaluated, as even the seemingly excessive protein intake in the diets of developed Western countries cannot prevent a reversal of the height trend. The promotion of a ‘sustainable’ plant-based diet for children, motivated by ecological rather than scientific reasons, does not sufficiently respect the specific requirements of this population group and will lead to suboptimal consumption of the necessary nutrients with the risk of permanent psychological and physical consequences. On the contrary, the existence of an inverse relationship between height and obesity in developed countries shows that the prevalence of childhood obesity could be significantly suppressed by quality animal-based nutrition during the period of physical growth, with a potentially persistent effect in adulthood. The presented data also support the urgent need to revise the current nutritional recommendations for adults, which are based on very weak observational evidence that fails to be confirmed in controlled clinical trials and observational studies in non-Western populations. In particular, the claim that the ongoing epidemic of obesity and type 2 diabetes is stimulated by the consumption of animal products, especially red meat, cannot be reconciled with the elementary fact that the average height in the affected countries is decreasing, thus revealing a decreasing consumption of quality animal proteins.

In light of these findings, it is clear that government nutrition policies must be experimental data-driven and it is necessary to promote a revision of all clinical and cohort study findings, rearranging and reexamining the results to understand to what extent the correlations observed in ecological analyses are consistent with these and vice versa. Some of the experiences of our university team show that even on the basis of observational data, reliable results can be obtained if the questionnaires used are appropriately designed and formulated, or if populations with diametrically different eating habits are compared. Another very valuable methodological approach can be the analysis of long-term nutritional trends and the annual incidence of diseases within individual countries, the results of which should theoretically be consistent with ecological data based on a mass comparison of many countries. New competencies acquired in this way would enable a better understanding of dietary habits and public health nutrition management.

## Supplementary Material

Supplementary_Figures_LEGENDS.docx

Supplementary_Figures.docx

## Data Availability

The datasets used and/or analyzed during the present study are available from the corresponding author upon reasonable request.

## References

[1] Viroli G, Kalmpourtzidou A, Cena H. Exploring benefits and barriers of plant-based diets: health, environmental impact, food accessibility and acceptability. Nutrients. 2023;15(22):4723. doi: 10.3390/nu15224723. 38004117 PMC10675717

[2] The Planetary Health Diet. 2023 https://eatforum.org/eat-lancet-commission/the-planetary-health-diet-and-you/.

[3] Food For L. Schools and early years. 2024 https://www.foodforlife.org.uk/schools-and-early-years.

[4] State of the Nation, Children’s food in England. 2023, 2019. https://www.soilassociation.org/media/20412/state-of-the-nation_2019.pdf.

[5] National Child Measurement Programme, Provisional 2021/22 School Year Outputs. 2023. https://digital.nhs.uk/data-and-information/publications/statistical/national-child-measurement-programme/2023-24-school-year/age.

[6] Ljungvall Å, Zimmerman FJ. Bigger bodies:long-term trends and disparities in obesity and body-mass index among US adults, 1960–2008. Soc Sci Med. 2012;75(1):109–119. doi: 10.1016/j.socscimed.2012.03.003. 22551821

[7] OECD iLibrary. Health at a Glance; 2023. https://www.oecd-ilibrary.org/social-issues-migration-health/health-at-a-glance_19991312.

[8] Centers for Disease Control and Prevention (CDC.gov). https://www.cdc.gov/nchs/data/databriefs/db364-tables-508.pdf

[9] Archer E, Pavela G, Lavie CJ. The inadmissibility of what we eat in America and NHANES dietary data in nutrition and obesity research and the scientific formulation of national dietary guidelines. Mayo Clinic Proceed. 2015; 90(7):911–926. 10.1016/j.mayocp.2015.04.009 PMC4527547 26071068

[10] Nissen SE. US dietary guidelines: an evidence-free zone. Ann Intern Med. 2016;164(8):558–559. doi: 10.7326/M16-0035. 26783992

[11] Mente A, de Koning L, Shannon HS, et al. A systematic review of the evidence supporting a causal link between dietary factors and coronary heart disease. Arch Intern Med. 2009;169(7):659–669. doi: 10.1001/archinternmed.2009.38. 19364995

[12] Alexander DD. No association between meat intake and mortality in Asian countries. Am J Clin Nutr. 2013;98(4):865–866. doi: 10.3945/ajcn.113.072017. 24004896

[13] Wang X, Lin X, Ouyang YY, et al. Red and processed meat consumption and mortality: dose–response meta-analysis of prospective cohort studies. Public Health Nutr. 2016;19(5):893–905. doi: 10.1017/S1368980015002062. 26143683 PMC10270853

[14] FAOSTAT. Food balances. 2023 https://www.fao.org/faostat/en/#data/FBS (2010-), https://www.fao.org/faostat/en/#data/FBSH. (-2013).

[15] Key differences between new and old Food Balance Sheet (FBS) methodology. 2023. https://fenixservices.fao.org/faostat/static/documents/FBS/Key%20differences%20between%20new%20and%20old%20FBS%20June2022%20.pdf

[16] Vonderschmidt A, Arendarczyk B, Jaacks LM, et al. Analysis combining the multiple FAO food balance sheet datasets needs careful treatment. Lancet Planet Health. 2024;8(2):e69-71–e71. doi: 10.1016/S2542-5196(23)00276-0. 38331531

[17] Grasgruber P, Sebera M, Hrazdira E, et al. Food consumption and the actual statistics of cardiovascular diseases: an epidemiological comparison of 42 European countries. Food Nutr Res. 2016;60(1):31694. doi: 10.3402/fnr.v60.31694. 27680091 PMC5040825

[18] Mensink RP, Zock PL, Kester AD, et al. Effects of dietary fatty acids and carbohydrates on the ratio of serum total to HDL cholesterol and on serum lipids and apolipoproteins: a meta-analysis of 60 controlled trials. Am J Clin Nutr. 2003;77(5):1146–1155. doi: 10.1093/ajcn/77.5.1146. 12716665

[19] Grasgruber P, Hrazdira E, Sebera M, et al. Cancer incidence in Europe: an ecological analysis of nutritional and other environmental factors. Front Oncol. 2018;8:151. doi: 10.3389/fonc.2018.00151. 29951370 PMC6008386

[20] Thar CM, Jackson R, Swinburn B, et al. A review of the uses and reliability of food balance sheets in health research. Nutr Rev. 2020;78(12):989–1000. doi: 10.1093/nutrit/nuaa023. 32556245

[21] Walrabenstein W, de Jonge CS, Kretova AM, et al. Commentary: United States dietary trends since 1800: lack of association between saturated fatty acid consumption and non-communicable diseases. Front Nutr. 2022;9:891792. doi: 10.3389/fnut.2022.891792. 35571907 PMC9096699

[22] Rose G. Sick individuals and sick populations. Int J Epidemiol. 2001;30(3):427–432. doi: 10.1093/ije/30.3.427. 11416056

[23] Bajracharya R, Kaaks R, Katzke V. Food sources of animal protein in relation to overall and cause-specific mortality—causal associations or confounding? an analysis of the EPIC-heidelberg cohort. Nutrients. 2023;15(15):3322. doi: 10.3390/nu15153322. 37571259 PMC10421322

[24] Astrup A, Magkos F, Bier DM, et al. Saturated fats and health: a reassessment and proposal for food-based recommendations: JACC state-of-the-art review. J Am Coll Cardiol. 2020;76(7):844–857. doi: 10.1016/j.jacc.2020.05.077. 32562735

[25] Heileson JL. Dietary saturated fat and heart disease:a narrative review. Nutr Rev. 2020;78(6):474–485. doi: 10.1093/nutrit/nuz091. 31841151

[26] Valk R, Hammill J, Grip J. Saturated fat: villain and bogeyman in the development of cardiovascular disease? Eur J Prev Cardiol. 2022;29(18):2312–2321. doi: 10.1093/eurjpc/zwac194. 36059207

[27] Dong JY, Zhang YH, Wang P, et al. Meta-analysis of dietary glycemic load and glycemic index in relation to risk of coronary heart disease. Am J Cardiol. 2012;109(11):1608–1613. doi: 10.1016/j.amjcard.2012.01.385. 22440121

[28] Fan J, Song Y, Wang Y, et al. Dietary glycemic index, glycemic load, and risk of coronary heart disease, stroke, and stroke mortality: a systematic review with meta-analysis. PLoS One. 2012;7(12):e52182. doi: 10.1371/journal.pone.0052182. 23284926 PMC3527433

[29] Livesey G, Livesey H. Coronary heart disease and dietary carbohydrate, glycemic index, and glycemic load: dose-response meta-analyses of prospective cohort studies. Mayo Clinic Proceed Innov Qual Outcomes. 2019;3(1):52–69. 10.1016/j.mayocpiqo.2018.12.007 PMC6410335 30899909

[30] Jayedi A, Soltani S, Jenkins D, et al. Dietary glycemic index, glycemic load, and chronic disease: an umbrella review of meta-analyses of prospective cohort studies. Crit Rev Food Sci Nutr. 2022;62(9):2460–2469. doi: 10.1080/10408398.2020.1854168. 33261511

[31] Miller V, Micha R, Choi E, et al. Evaluation of the quality of evidence of the association of foods and nutrients with cardiovascular disease and diabetes: a systematic review. JAMA Netw Open. 2022;5(2):e2146705-e2146705. doi: 10.1001/jamanetworkopen.2021.46705. 35113165 PMC8814912

[32] Taubes G. The soft science of dietary fat. Science. 2001;291(5513):2536–2545. doi,. doi: 10.1126/science.291.5513.2536. 11286266

[33] Harcombe Z. US dietary guidelines:is saturated fat a nutrient of concern? Br J Sports Med. 2019;53(22):1393–1396. doi: 10.1136/bjsports-2018-099420. 30108061

[34] Harcombe Z, Baker JS, Cooper SM, et al. Evidence from randomised controlled trials did not support the introduction of dietary fat guidelines in 1977 and 1983: a systematic review and meta-analysis. Open Heart. 2015;2(1):e000196. doi: 10.1136/openhrt-2014-000196. 25685363 PMC4316589

[35] Menotti A, Keys A, Blackburn H, et al. Twenty-year stroke mortality and prediction in twelve cohorts of the Seven Countries Study. Int J Epidemiol. 1990;19(2):309–315. doi: 10.1093/ije/19.2.309. 2198235

[36] Johnston B, De Smet S, Leroy F, et al. Non-communicable disease risk associated with red and processed meat consumption—magnitude, certainty, and contextuality of risk? Anim Front. 2023;13(2):19–27. doi: 10.1093/af/vfac095. 37073320 PMC10105855

[37] Guasch-Ferré M, Satija A, Blondin SA, et al. Meta-analysis of randomized controlled trials of red meat consumption in comparison with various comparison diets on cardiovascular risk factors. Circulation. 2019;139(15):1828–1845. doi: 10.1161/CIRCULATIONAHA.118.035225. 30958719

[38] Sanders LM, Palacios OM, Wilcox ML, et al. Beef consumption and cardiovascular risk factors: a systematic review and meta-analysis of randomized controlled trials. Curr Dev Nutr. 2024;8(12):104500. doi: 10.1016/j.cdnut.2024.104500. 39649475 PMC11621491

[39] Fontecha J, Calvo MV, Juarez M, et al. Milk and dairy product consumption and cardiovascular diseases: an overview of systematic reviews and meta-analyses. Adv Nutr. 2019;10(suppl_2):S164–S189. doi: 10.1093/advances/nmy099. 31089735 PMC6518146

[40] Dehghan M, Mente A, Zhang X, et al. Associations of fats and carbohydrate intake with cardiovascular disease and mortality in 18 countries from five continents (PURE): a prospective cohort study. Lancet. 2017;390(10107):2050–2062. doi: 10.1016/S0140-6736(17)32252-3. 28864332

[41] Mente A, Dehghan M, Rangarajan S, et al. Diet, cardiovascular disease, and mortality in 80 countries. Eur Heart J. 2023;44(28):2560–2579. doi: 10.1093/eurheartj/ehad269. 37414411 PMC10361015

[42] Gianos E, Williams KA, Freeman AM, et al. How pure is PURE? Dietary lessons learned and not learned from the PURE Trials. Am J Med. 2018;131(5):457–458. doi: 10.1016/j.amjmed.2017.11.024. 29229470

[43] Beal T, Ortenzi F, Fanzo J. Estimated micronutrient shortfalls of the EAT–Lancet planetary health diet. Lancet Planet Health. 2023;7(3):e233-237–e237. doi: 10.1016/S2542-5196(23)00006-2. 36889864

[44] Tuomisto HL. The complexity of sustainable diets. Nat Ecol Evol. 2019;3(5):720–721. doi: 10.1038/s41559-019-0875-5. 30988495

[45] Levine ME, Suarez JA, Brandhorst S, et al. Low protein intake is associated with a major reduction in IGF-1, cancer, and overall mortality in the 65 and younger but not older population. Cell Metab. 2014;19(3):407–417. doi: 10.1016/j.cmet.2014.02.006. 24606898 PMC3988204

[46] Gu X, Drouin-Chartier JP, Sacks FM, et al. Red meat intake and risk of type 2 diabetes in a prospective cohort study of United States females and males. Am J Clin Nutr. 2023;118(6):1153–1163. doi: 10.1016/j.ajcnut.2023.08.021. 38044023 PMC10739777

[47] Bao J, Atkinson F, Petocz P, et al. Prediction of postprandial glycemia and insulinemia in lean, young, healthy adults: glycemic load compared with carbohydrate content alone. Am J Clin Nutr. 2011;93(5):984–996. doi: 10.3945/ajcn.110.005033. 21325437

[48] Miller V, Jenkins DA, Dehghan M, et al. Associations of the glycaemic index and the glycaemic load with risk of type 2 diabetes in 127 594 people from 20 countries (PURE): a prospective cohort study. Lancet Diabetes Endocrinol. 2024;12(5):330–338. doi: 10.1016/S2213-8587(24)00069-X. 38588684

[49] Neuenschwander M, Ballon A, Weber KS, et al. Role of diet in type 2 diabetes incidence: umbrella review of meta-analyses of prospective observational studies. BMJ. 2019;366:l2368. doi: 10.1136/bmj.l2368. 31270064 PMC6607211

[50] Toi PL, Anothaisintawee T, Chaikledkaew U, et al. Preventive role of diet interventions and dietary factors in type 2 diabetes mellitus: an umbrella review. Nutrients. 2020;12(9):2722. doi: 10.3390/nu12092722. 32899917 PMC7551929

[51] DiNicolantonio JJ, O’Keefe JH, Lucan SC. Added fructose: a principal driver of type 2 diabetes mellitus and its consequences. Mayo Clin Proc. 2015;90(3):372–381. doi: 10.1016/j.mayocp.2014.12.019. 25639270

[52] Bentley RA, Ruck DJ, Fouts HN. US obesity as delayed effect of excess sugar. Economics & Human Biology. 2020;36:100818. doi: 10.1016/j.ehb.2019.100818. 31540873

[53] Lee JH, Duster M, Roberts T, et al. United States dietary trends since 1800: lack of association between saturated fatty acid consumption and non-communicable diseases. Front Nutr. 2021;8:748847. doi: 10.3389/fnut.2021.748847. 35118102 PMC8805510

[54] Wiegers C, van de Burgwal LH, Claassen E, et al. Trends in nutrition, lifestyle, and metabolic disease in the United States from 1900 onwards. PharmaNutrition. 2023;25:100350.

[55] Seiquer I, Díaz-Alguacil J, Delgado-Andrade C, et al. Diets rich in Maillard reaction products affect protein digestibility in adolescent males aged 11–14 y. Am J Clin Nutr. 2006;83(5):1082–1088. doi: 10.1093/ajcn/83.5.1082. 16685050

[56] Dyck R, Osgood N, Lin TH, et al. Epidemiology of diabetes mellitus among First Nations and non-First Nations adults. CMAJ. 2010;182(3):249–256. doi: 10.1503/cmaj.090846. 20083562 PMC2826466

[57] Carstensen B, Rønn PF, Jørgensen ME. Prevalence, incidence and mortality of type 1 and type 2 diabetes in Denmark 1996-2016. BMJ Open Diabetes Res Care. 2020;8(1):e001071. Electronic Supplementary material, Table ESM 4.”. Both articles use the same data but only this article has them accessible in the Supplementary material. doi: 10.1136/bmjdrc-2019-001071. PMC7265004 32475839

[58] de Sousa-Uva M, Antunes L, Nunes B, et al. Trends in diabetes incidence from 1992 to 2015 and projections for 2024: a Portuguese General Practitioner’s Network study. Prim Care Diabetes. 2016;10(5):329–333. doi: 10.1016/j.pcd.2016.05.003. 27363730

[59] Holden SE, Barnett AH, Peters JR, et al. The incidence of type 2 diabetes in the United Kingdom from 1991 to 2010. Diabetes Obes Metab. 2013;15(9):844–852. doi: 10.1111/dom.12123. 23675742

[60] Etemadi A, Sinha R, Ward MH, et al. Mortality from different causes associated with meat, heme iron, nitrates, and nitrites in the NIH-AARP Diet and Health Study: population based cohort study. BMJ. 2017;357:j1957. doi: 10.1136/bmj.j1957. 28487287 PMC5423547

[61] Viegi G, Maio S, Fasola S, et al. Global burden of chronic respiratory diseases. J Aerosol Med Pulm Drug Deliv. 2020;33(4):171–177. doi: 10.1089/jamp.2019.1576. 32423274

[62] Cheemerla S, Balakrishnan M. Global epidemiology of chronic liver disease. Clin Liver Dis (Hoboken). 2021;17(5):365–370. doi: 10.1002/cld.1061. 34136143 PMC8177826

[63] Sinha R, Cross AJ, Graubard BI, et al. Meat intake and mortality: a prospective study of over half a million people. Arch Intern Med. 2009;169(6):562–571. doi: 10.1001/archinternmed.2009.6. 19307518 PMC2803089

[64] Song M, Fung TT, Hu FB, et al. Association of animal and plant protein intake with all-cause and cause-specific mortality. JAMA Intern Med. 2016;176(10):1453–1463. doi: 10.1001/jamainternmed.2016.4182. 27479196 PMC5048552

[65] Kim Y, Keogh J, Clifton P. A review of potential metabolic etiologies of the observed association between red meat consumption and development of type 2 diabetes mellitus. Metabolism. 2015;64(7):768–779. doi: 10.1016/j.metabol.2015.03.008. 25838035

[66] Fretts AM, Follis JL, Nettleton JA, et al. Consumption of meat is associated with higher fasting glucose and insulin concentrations regardless of glucose and insulin genetic risk scores: a meta-analysis of 50,345 Caucasians. Am J Clin Nutr. 2015;102(5):1266–1278. doi: 10.3945/ajcn.114.101238. 26354543 PMC4625584

[67] O’Connor LE, Kim JE, Clark CM, et al. Milk and dairy product consumption and cardiovascular diseases: an overview of systematic reviews and meta-analyses. Adv Nutr. 2021;12(1):115–127. doi: 10.1093/advances/nmaa096. 31089735 PMC6518146

[68] Sanders LM, Wilcox ML, Maki KC. Red meat consumption and risk factors for type 2 diabetes: a systematic review and meta-analysis of randomized controlled trials. Eur J Clin Nutr. 2023;77(2):156–165. doi: 10.1038/s41430-022-01150-1. 35513448 PMC9908545

[69] FAO of the United Nations. Dietary protein quality evaluation in human nutrition. Report of an FAO Expert Consultation. 2013. 26369006

[70] Leser S. The 2013 FAO report on dietary protein quality evaluation in human nutrition: recommendations and implications. Nutr Bull. 2013;38(4):421–428. doi: 10.1111/nbu.12063.

[71] Adhikari S, Schop M, de Boer IJ, et al. Protein quality in perspective: a review of protein quality metrics and their applications. Nutrients. 2022;14(5):947. doi: 10.3390/nu14050947. 35267922 PMC8912699

[72] Marinangeli CP. Complementing cereal grains with pulse grains to enhance the nutritional and environmental sustainability profiles of manufactured foods in Canada and the United States. Cereal Foods World. 2020;65(6):63. doi: 10.1094/CFW-65-6-0063.

[73] Nutridatabaze.cz. https://www.nutridatabaze.cz/potraviny/?id=497. (Peas, boiled in unsalted water), https://www.nutridatabaze.cz/potraviny/?id=504. (Rice, boiled in unsalted Rice white water), https://www.nutridatabaze.cz/potraviny/?id=281. (Pork, loin, boneless, separable lean only, roasted).

[74] Grasgruber P, Hrazdíra E. Nutritional and socio-economic predictors of adult height in 152 world populations. Econ Hum Biol. 2020;37:100848. doi: 10.1016/j.ehb.2020.100848. 32247188

[75] Börnhorst C, Huybrechts I, Hebestreit A, et al. Usual energy and macronutrient intakes in 2–9-year-old European children. Int J Obes. 2014;38(S2):S115–S123.) doi: 10.1038/ijo.2014.142. 25376213

[76] Garcia-Iborra M, Castanys-Munoz E, Oliveros E, et al. Optimal protein intake in healthy children and adolescents: evaluating current evidence. Nutrients. 2023;15(7):1683. doi: 10.3390/nu15071683. 37049523 PMC10097334

[77] Elango R, Humayun MA, Ball RO, et al. Protein requirement of healthy school-age children determined by the indicator amino acid oxidation method. Am J Clin Nutr. 2011;94(6):1545–1552. doi: 10.3945/ajcn.111.012815. 22049165

[78] Grasgruber P, Hrazdíra E, Hlavoňová Z, et al. Body height, body composition, and lifestyle of Czech high school students: implications for the most appropriate strategies promoting physical growth and preventing obesity. J Physiol Anthropol. in press. 10.1186/s40101-025-00418-2 PMC13045034 41796343

[79] Grasgruber P, Popović S, Bokuvka D, et al. The mountains of giants: an anthropometric survey of male youths in Bosnia and Herzegovina. R Soc Open Sci. 2017;4(4):161054. doi: 10.1098/rsos.161054. 28484621 PMC5414258

[80] Segovia-Siapco G, Khayef G, Pribis P, et al. Animal protein intake is associated with general adiposity in adolescents: the teen food and development study. Nutrients. 2019;12(1):110. doi: 10.3390/nu12010110. 31906138 PMC7019331

[81] Johnson W, Stovitz SD, Choh AC, et al. Patterns of linear growth and skeletal maturation from birth to 18 years of age in overweight young adults. Int J Obes (Lond). 2012;36(4):535–541. doi: 10.1038/ijo.2011.238. 22124455 PMC3312969

[82] Health Survey for England 2021. 2023. https://files.digital.nhs.uk/03/D7F19D/HSE-2021-Overweight-and-obesity-tables.xlsx (Table 6).

[83] National Center for Health Statistics. https://www.cdc.gov/. Anthropometric Reference Data for Children and Adults, United States, 1988-1994. 2023. https://www.cdc.gov/nchs/data/series/sr_11/sr11_249.pdf, Anthropometric Reference Data for Children and Adults, U.S. Population, 1999–2002. https://www.cdc.gov/nchs/data/ad/ad361.pdf, Anthropometric Reference Data for Children and Adults, United States, 2003–2006. https://www.cdc.gov/nchs/data/nhsr/nhsr010.pdf, Anthropometric Reference Data for Children and Adults, United States, 2007–2010, https://www.cdc.gov/nchs/data/series/sr_11/sr11_252.pdf, Anthropometric Reference Data for Children and Adults, United States, 2011–2014. https://www.cdc.gov/nchs/data/series/sr_03/sr03_039.pdf, Anthropometric Reference Data for Children and Adults, United States, 2015–2018. https://www.cdc.gov/nchs/data/series/sr_03/sr03-046-508.pdf

[84] Shomaker LB, Tanofsky-Kraff M, Savastano DM, et al. Puberty and observed energy intake: boy, can they eat!. Am J Clin Nutr. 2010;92(1):123–129. doi: 10.3945/ajcn.2010.29383. 20504975 PMC2884323

[85] Office for National Statistics. Census. 2001. https://webarchive.nationalarchives.gov.uk/…ethnic-group.xls. Census 2011. https://webarchive.nationalarchives.gov.uk/…rft-ks201uk.xls. Census 2021. https://www.ons.gov.uk/datasets/…ethnic_group_tb_20b

[86] The Migration Observatory. Citizenship and naturalisation for migrants in the UK. 2023. https://migrationobservatory.ox.ac.uk/resources/briefings/citizenship-and-naturalisation-for-migrants-in-the-uk/. The Migration Observatory. 2024. Migrants in the UK: An Overview. https://migrationobservatory.ox.ac.uk/resources/briefings/migrants-in-the-uk-an-overview/

[87] Hispanic or Latino Origin by Race: 2010 and 2020. https://www2.census.gov/programs-surveys/decennial/2020/data/redistricting-supplementary-tables/redistricting-supplementary-table-04.pdf.

[88] Pew Research Center. Key findings about Black immigrants in the U.S. 2022. https://www.pewresearch.org/short-reads/2022/01/27/key-findings-about-black-immigrants-in-the-u-s/.

[89] Schönbeck Y, Talma H, van Dommelen P, et al. The world’s tallest nation has stopped growing taller: the height of Dutch children from 1955 to 2009. Pediatr Res. 2013;73(3):371–377. doi: 10.1038/pr.2012.189. 23222908

[90] Kromhout D, Spaaij CJ, de Goede J, et al. The 2015 Dutch food-based dietary guidelines. Eur J Clin Nutr. 2016;70(8):869–878. doi: 10.1038/ejcn.2016.52. 27049034 PMC5399142

[91] Wit JA, Peters S. Nederlanders zijn de langste mensen ter wereld, maar we groeien niet meer (The Dutch are the tallest people in the world, but they’ve stopped growing). Voeding Magazine. 2019;1:20–23. https://www.zuivelengezondheid.nl/wp-content/uploads/2019/04/Voeding-Magazine-1-2019-NW.pdf. (translation from Dutch: https://www.researchgate.net/publication/332442247_Height_in_The_Netherlands_The_Dutch_are_the_tallest_people_in_the_world_but_they%27ve_stopped_growing.)

[92] Centraal Bureau voor de Statistiek. Lichaamslengte. 2021. https://www.cbs.nl/nl-nl/maatwerk/2021/37/lichaamslengte

[93] Krul AJ, Daanen HA, Choi H. Self-reported and measured weight, height and body mass index (BMI) in Italy, the Netherlands and North America. Eur J Public Health. 2011;21(4):414–419. doi: 10.1093/eurpub/ckp228. 20089678

[94] Zehetmayer M. The continuation of the antebellum puzzle: stature in the US, 1847–1894. Europ Rev Econ Hist. 2011;15(2):313–327. doi: 10.1017/S1361491611000062.

[95] Heyberger L. (2012). New anthropometric history: An analysis of the secular trend in height. In: Preedy VR, editor. Handbook of Anthropometry, Physical Measures of Human Form in Health and Disease. Springer Science & Business Media, p. 253–270.

[96] Komlos J. Anthropometric history: an overview of a quarter century of research. Anthropol Anz. 2009;67(4):341–356. doi: 10.1127/0003-5548/2009/0027. 20440956

[97] Gleiss A, Lassi M, Blümel P, et al. Austrian height and body proportion references for children aged 4 to under 19 years. Ann Hum Biol. 2013;40(4):324–332. doi: 10.3109/03014460.2013.776110. 23590681

[98] Kirchengast S, Juan A, Waldhoer T, et al. An increase in the developmental tempo affects the secular trend in height in male Austrian conscripts birth cohorts 1951–2002. Am J Hum Biol. 2023;35(4):e23848. doi: 10.1002/ajhb.23848. 36510339

[99] Hatton TJ. How have Europeans grown so tall? Oxford Economic Papers. 2014;66(2):349–372. doi: 10.1093/oep/gpt030.

[100] The World Bank. 2023. https://data.worldbank.org/indicator.

[101] Human Development Reports. The Human Development Index. 2023. https://hdr.undp.org/data-center/human-development-index#/indicies/HDI.

[102] US Census Bureau. Income in the United States. 2022. https://www.census.gov/library/publications/2023/demo/p60-279.html.

[103] Ministry of Defence, Denmark. Statistiske oplysniniger.Udfaldet, gennemsnitshøjden og BMI på forsvarets dag. (Statistical information. Results, average height, and BMI on the Defense Day) 2021. 2023. https://www.forpers.dk/globalassets/fps/dokumenter/2021/-statistik-forsvarets-dag-sep-2021-.pdf.

[104] The Finnish Defence Forces (M. Santtila – personal communication). 2024.

[105] Statistics Norway (Statistik sentralbyrå). Tabell 4.22. Vernepliktige, etter høyde. Prosent. 2023 [Conscripts, by height. Percentage]. https://www.ssb.no/a/histstat/tabeller/4-22.html;. Egenrapporterthøydeogvekt for sesjonspliktige (SÅ 108), https://www.ssb.no/helse/helseforhold-og-levevaner/statistikk/helseforhold-levekarsundersokelsen

[106] Statistical Yearbook (Statistiskårbok). 2023 https://www.ssb.no/befolkning/artikler-og-publikasjoner/statistisk-aarbok.

[107] Ludvigsson JF, Berglind D, Sundquist K, et al. The Swedish military conscription register: opportunities for its use in medical research. Eur J Epidemiol. 2022;37(7):767–777. doi: 10.1007/s10654-022-00887-0. 35810240 PMC9329412

[108] Sjöberg A, Barrenäs M-L, Brann E, et al. Body size and lifestyle in an urban population entering adulthood: the ‘Grow up Gothenburg’ study. Acta Paediatr. 2012;101(9):964–972. doi: 10.1111/j.1651-2227.2012.02722.x. 22577752

[109] Lehmann A, Floris J, Woitek U, et al. Temporal trends, regional variation and socio-economic differences in height, BMI and body proportions among German conscripts, 1956–2010. Public Health Nutr. 2017;20(3):391–403. doi: 10.1017/S1368980016002408. 27629891 PMC10261587

[110] Statistisches Bundesamt. Press: 24.3% of the population had a history of immigration in 2022. https://www.destatis.de/EN/Press/2023/04/PE23_158_125.html

[111] Bundesamt S. Presse: 15% mehr Einbürgerungen im Jahr. 2019. https://www.destatis.de/DE/Presse/Pressemitteilungen/2020/06/PD20_197_12511.html-.

[112] McLaren L. Socio-economic status and obesity. Epidemiol Rev. 2007;29(1):29–48. doi: 10.1093/epirev/mxm001. 17478442

[113] Barriuso L, Miqueleiz E, Albaladejo R, et al. Socio-economic position and childhood-adolescent weight status in rich countries: a systematic review, 1990–2013. BMC Pediatr. 2015;15(1):129. doi: 10.1186/s12887-015-0443-3. 26391227 PMC4578240

[114] Diverse Populations Collaborative Group. Weight‐height relationships and body mass index: some observations from the diverse populations collaboration. Am J Phys Anthropol. 2005;128(1):220–229. doi: 10.1002/ajpa.20107. 15761809

[115] The World Health Organization: The Global Health Observatory. Prevalence of obesity among children and adolescents, BMI > +2 standard deviations above the median (crude estimate) (%). https://www.who.int/data/gho/data/indicators/indicator-details/GHO/prevalence-of-obesity-among-children-and-adolescents-bmi-2-standard-deviations-above-the-median-(crude-estimate)-(-).

[116] Sichieri R, dos Santos Barbosa F, Moura EC. Relationship between short stature and obesity in Brazil: a multilevel analysis. Br J Nutr. 2010;103(10):1534–1538. doi: 10.1017/S0007114509993448. 20070916

[117] Rickenbacher M, Gültekin N, Stanga Z, et al. The role of body height as a co‐factor of excess weight in Switzerland. Am J Hum Biol. 2022;34(8):e23754. doi: 10.1002/ajhb.23754. 35488790 PMC9541525

[118] Cohen DA, Sturm R. Body mass index is increasing faster among taller persons. Am J Clin Nutr. 2008;87(2):445–448. doi: 10.1093/ajcn/87.2.445. 18258637

[119] Simonson M, Boirie Y, Guillet C. Protein, amino acids and obesity treatment. Rev Endocr Metab Disord. 2020;21(3):341–353. doi: 10.1007/s11154-020-09574-5. 32827096 PMC7455583

[120] Consultation FE. Dietary protein quality evaluation in human nutrition. FAO Food and Nutrition Paper. 2011;31:1–66, 92. 26369006

[121] Pereira AR, Oliveira A. Dietary interventions to prevent childhood obesity: a literature review. Nutrients. 2021;13(10):3447. doi: 10.3390/nu13103447. 34684448 PMC8537925

[122] Kahleova H, Petersen KF, Shulman GI, et al. Effect of a low-fat vegan diet on body weight, insulin sensitivity, postprandial metabolism, and intramyocellular and hepatocellular lipid levels in overweight adults: a randomized clinical trial. JAMA Netw Open. 2020;3(11):e2025454. doi: 10.1001/jamanetworkopen.2020.25454. 33252690 PMC7705596

[123] Flynn JR. Requiem for nutrition as the cause of IQ gains: raven’s gains in Britain 1938–2008. Econ Hum Biol. 2009;7(1):18–27. doi: 10.1016/j.ehb.2009.01.009. 19251490

[124] Grantham-McGregor S, Cheung YB, Cueto S, et al. Developmental potential in the first 5 years for children in developing countries. Lancet. 2007;369(9555):60–70. doi: 10.1016/S0140-6736(07)60032-4. 17208643 PMC2270351

[125] Case A, Paxson C. Causes and consequences of early-life health. Demography. 2010;47 Suppl(Suppl 1):S65–S85. doi: 10.1353/dem.2010.0007. 21302429 PMC3730845

[126] Spears D. Height and cognitive achievement among Indian children. Econ Hum Biol. 2012;10(2):210–219. doi: 10.1016/j.ehb.2011.08.005. 21907646

[127] Christensen GT, Molbo D, Ängquist LH, et al. Cohort profile, The Danish Conscription Database (DCD): a cohort of 728 160 men born from 1939 through 1959. Int J Epidemiol. 2015;44(2):432–440. doi: 10.1093/ije/dyu114. 24906367

[128] Walker SP, Wachs TD, Gardner JM, et al. Child development, risk factors for adverse outcomes in developing countries. The. Lancet. 2007;369(9556):145–157. doi: 10.1016/S0140-6736(07)60076-2. 17223478

[129] Nyaradi A, Li J, Hickling S, et al. The role of nutrition in children’s neurocognitive development, from pregnancy through childhood. Front Hum Neurosci. 2013;7:97. doi: 10.3389/fnhum.2013.00097. 23532379 PMC3607807

[130] Gunnell D, Miller LL, Rogers I, ALSPAC Study Team., et al. Association of insulin-like growth factor I and insulin-like growth factor–binding protein-3 with intelligence quotient among 8-to 9-year-old children in the Avon Longitudinal Study of Parents and Children. Pediatrics. 2005;116(5):e681–e686. doi: 10.1542/peds.2004-2390. 16263982

[131] Taki Y, Hashizume H, Sassa Y, et al. Correlation among body height, intelligence, and brain gray matter volume in healthy children. Neuroimage. 2012;59(2):1023–1027. doi: 10.1016/j.neuroimage.2011.08.092. 21930215

[132] Hulett JL, Weiss RE, Bwibo NO, et al. Relationship between short stature and obesity in Brazil: a multilevel analysis. Br J Nutr. 2014;111(5):875–886. doi: 10.1017/S0007114513003310. 24168874

[133] Borge TC, Aase H, Brantsæter AL, et al. The importance of maternal diet quality during pregnancy on cognitive and behavioural outcomes in children, a systematic review and meta-analysis. BMJ Open. 2017;7(9):e016777. doi: 10.1136/bmjopen-2017-016777. PMC5623570 28947450

[134] Dutton E, van der Linden D, Lynn R. The negative Flynn Effect: A systematic literature review. Intelligence. 2016;59:163–169. doi: 10.1016/j.intell.2016.10.002.

[135] Dworak EM, Revelle W, Condon DM. Looking for Flynn effects in a recent online US adult sample: examining shifts within the SAPA Project. Intelligence. 2023;98:101734. doi: 10.1016/j.intell.2023.101734.

[136] Bratsberg B, Rogeberg O. Flynn effect and its reversal are both environmentally caused. Proc Natl Acad Sci U S A. 2018;115(26):6674–6678. doi: 10.1073/pnas.1718793115. 29891660 PMC6042097

[137] Hegelund ER, Teasdale TW, Okholm GT, et al. The secular trend of intelligence test scores: the Danish experience for young men born between 1940 and 2000. PLoS One. 2021;16(12):e0261117. doi: 10.1371/journal.pone.0261117. 34882746 PMC8659667

[138] Dutton E, Lynn R. A negative Flynn effect in Finland, 1997–2009. Intelligence. 2013;41(6):817–820. doi: 10.1016/j.intell.2013.05.008.

[139] Neumann CG, Murphy SP, Gewa C, et al. Meat Supplementation Improves Growth, Cognitive, and Behavioral Outcomes in Kenyan Children. J Nutr. 2007;137(4):1119–1123. doi: 10.1093/jn/137.4.1119. 17374691

