# Correction

**DOI:** 10.1080/07853890.2025.2535823

**Published:** 2025-07-21

**Article title:** Back to the pre-industrial age? FAOSTAT statistics of food supply reveal radical dietary changes accompanied by declining body height, rising obesity rates, and declining phenotypic IQ in affluent Western countries

**Authors:** Pavel Grasgruber

**Journal:** *Annals of Medicine*

**Bibliometrics:** Volume 56, Number 01, page 2514073

**DOI:** https://doi.org/10.1080/07853890.2025.2514073

When the above article was published online, it contained incorrect values in the following sentences. These have been corrected and republished with accurate information.

1. The following sentence has been changed from ‘potatoes (*r* = 0.75, eight-year lag), lag), and starchy roots (*r* = 0.74, eight-year lag)’ to ‘potatoes (*r* = 0.74, eight-year lag), lag), and starchy roots (*r* = 0.73, eight-year lag)’
2. The following sentence has been changed from ‘between 1965–2021 (zero lag). In Canada (*r* = −0.64, six-year lag) and the United Kingdom (*r* = −0.50, seven-year lag) (*p* < 0.05), it is even stronger.’, to ‘between 1965–2021 (zero lag, p < 0.05). In Canada (r = −0.65, p < 0.05, six-year lag) and the United Kingdom (*r* = −0.50, p > 0.05, seven-year lag), r-values tend to be even higher.’
3. The following sentence has been changed from ‘we also observe that total energy intake from red meat (beef, mutton, pork) is significantly inversely related to diabetes incidence’ to ‘we also observe that total energy intake from red meat (beef, mutton, pork) is always inversely related to diabetes incidence’
4. The following sentence has been changed from ‘The association of the height trend with the protein index between 1970–2020 is evident, but according to the cross-correlation, the trend lines do not have exactly the same shape and reach significant similarity (*p* < 0.05) only in the combined sample (with a lag of 0–1 year for men and 0–3 years for women)’ to ‘The association of the height trend with the protein index is evident and when a maximum time lag of 19 years is used (in accordance with age), the strongest cross-correlation between these two variables can be observed in the aggregated samples of ethnic Dutch and 2^nd^ generation immigrants (r = 0.35 for men at a lag of three years, r = 0.44 for women at a lag of two years, p < 0.05,’
5. The following sentence has been changed from ‘not similar in terms of the cross-correlation analysis (*p* > 0.05)’ to ‘only moderately cross-correlated (r = 0.48, p < 0.05, zero lag)’
6. The following sentence has been changed from ‘Although there is again no long-term positive cross-correlation between the protein index and height’ to ‘Although there is no long-term positive cross-correlation between the protein index and height’
7. The following sentence has been changed from ‘which identifies significant similarities at lags of 0–10 years, with a peak at zero lag (*r* = 0.69).’ to ‘which identifies significant similarities at lags of 0–3 years, with a peak at a lag of three years (*r* = 0.52).’
8. The following sentence has been changed from ‘with a significant cross-correlation at lags of 0–5 years (with a peak of *r* = 0.88 at zero lag)’ to ‘with a significant cross-correlation at lags of 0–7 years (with a peak of *r* = 0.85 at zero lag)’.
9. The following sentence has been changes from ‘Norway between 1961–2011 shows an almost perfect linear correlation (*r* = 0.89, *p* < 0.001) with the combined consumption of four high-quality, non-dairy protein sources (beef, eggs, fish & seafood, pork), which is also confirmed by the cross-correlation (*r* = 0.93, *p* < 0.05 at zero lag) (Figure 8C)’ to ‘Norway between 1961–2011 shows an almost perfect linear correlation (*r* = 0.88, *p* < 0.001) with the combined consumption of four high-quality, non-dairy protein sources (beef, eggs, fish & seafood, pork), which is also confirmed by the cross-correlation (*r* = 0.85, *p* < 0.05 at zero lag) (Figure 8C)’.

