# Supplementary material for: Back to the pre-industrial age? FAOSTAT statistics of food supply reveal radical dietary changes accompanied by declining body height, rising obesity rates, and declining phenotypic IQ in affluent Western countries
Source: Ann Med. 2025 Jun 14;57(1):2514073. doi: 10.1080/07853890.2025.2514073 (PMC12168400; doi:10.1080/07853890.2025.2514073)
Supplement: Supplementary_Figures_LEGENDS.docx [file IANN_A_2514073_SM7354.docx]

**Supplementary Figure 1. A) Age-standardized diabetes incidence in adults (type 1 and 2) in Canada, Saskatchewan province (1980-2003) plotted against average per capita energy intake (kcal/day) of selected food items.** Data on food energy intake have been shifted forward in time, according to their strongest cross-correlation with diabetes incidence. The underlying analysis includes 26 food items with an average per capita energy intake of ≥ 50 kcal/day between 1965-2003. **B) Factor analysis of diabetes incidence in Canada, Saskatchewan province (1980-2003) and 26 food items with an average per capita energy intake of ≥ 50 kcal/day (1978-2001).** Data on food energy intake have been shifted two years forward in time. *Sources:* FAOSTAT: Food balances [14]; Dyck et al. [57].

**Supplementary Figure 2. A) Type 2 diabetes incidence in the United Kingdom (1991-2010) plotted against average per capita energy intake (kcal/day) of selected food items.** Data on food energy intake have been shifted forward in time, according to their strongest cross-correlation with type 2 diabetes incidence. The underlying analysis includes 26 food items: the combined consumption of carbohydrates from cereals and refined sugar & sweeteners, and 25 foods with an average per capita energy intake of ≥ 50 kcal/day between 1976-2010. **B) Factor analysis of type 2 diabetes incidence in the United Kingdom (1991-2010) and 26 food items with an average per capita energy intake of ≥ 50 kcal/day (1990-2009).** Data on food energy intake have been shifted one year forward in time. *Sources:* FAOSTAT: Food balances [14]; Holden et al. [60].

**Supplementary figure 3. Trends in protein consumption from main food items between 1961-2020.** *Source:* FAOSTAT: Food balances [14]. *Note:* Data for the period 1961-2013 are according to the older FAOSTAT methodology. Data for the period 2014-2020 are according to the new FAOSTAT methodology, but before the 2021 update and subsequent revision.

**Supplementary figure 4. Trends in protein consumption from main food items between 1961-2020.** *Source:* FAOSTAT: Food balances [14]. *Note:* Data for the period 1961-2013 are according to the older FAOSTAT methodology. Data for the period 2014-2020 are according to the new FAOSTAT methodology, but before the 2021 update and subsequent revision.

**Supplementary figure 5. Trends in protein consumption from main food items between 1961-2020.** *Source:* FAOSTAT: Food balances [14]. *Note:* Data for the period 1961-2013 are according to the older FAOSTAT methodology. Data for the period 2014-2020 are according to the new FAOSTAT methodology, but before the 2021 update and subsequent revision.
