# Supplementary material for: Back to the pre-industrial age? FAOSTAT statistics of food supply reveal radical dietary changes accompanied by declining body height, rising obesity rates, and declining phenotypic IQ in affluent Western countries
Source: Ann Med. 2025 Jun 14;57(1):2514073. doi: 10.1080/07853890.2025.2514073 (PMC12168400; doi:10.1080/07853890.2025.2514073)
Supplement: Supplementary_Figures.docx [file IANN_A_2514073_SM7353.docx]

**Supplementary Figures**

|  |  |
| --- | --- |
| **Supplementary Figure 1.** | |

|  |  |
| --- | --- |
| **Supplementary Figure 2.** | |

**
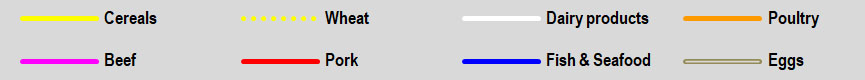
**

**Supplementary figure 3.**

**
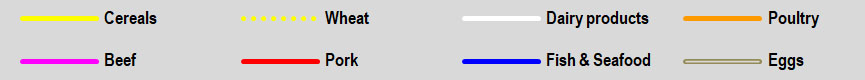
**

**Supplementary figure 4.**

**
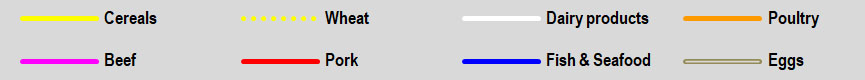
**

**Supplementary figure 5.**
